# Next-Generation Sequencing Dataset Downloader and In Silico Sequence Mining: Graphical-User-Interface-Based Tools for Accessible, Multiprobe Target Mining in Next-Generation Sequencing Data

**DOI:** 10.34133/csbj.0095

**Published:** 2026-05-14

**Authors:** Min Chan Kim, Hye Ji Jung, Min Chang Kang, Ha Yeon Kim, Seong Sik Jang, Alain Chrysler Chamfort, Han Byul Lee, Hye Won Bae, Dae Gwin Jeong, Hye Kwon Kim

**Affiliations:** ^1^ Department of Biological Sciences and Biotechnology, College of Natural Sciences, Chungbuk National University, Cheongju, Republic of Korea.; ^2^ Bionanotechnology Research Center, Korea Research Institute of Bioscience and Biotechnology, Daejeon, Korea.

## Abstract

Next-generation sequencing (NGS) is expanding in clinical practice, yet many pipelines defer actionable decisions until full alignment and taxonomic calling are complete. The primary contribution of In Silico Sequence Mining (ISSM) lies in providing an accessible graphical-user-interface-based workflow for rapid probe-based pre-alignment screening of raw FASTQ/FASTA datasets. ISSM performs probe-based matching of predefined targets directly on raw FASTQ/FASTA files and supports configurable match thresholds and subsampling. We assessed ISSM using 4 datasets, including 34,899 coronavirus genomes, NGS data from CRFK cells infected with feline coronavirus (FCoV), plasma-derived HIV datasets from patients with sepsis, and colorectal cancer sequencing datasets. Public primer/probe panels applied to coronavirus genomes reliably identified targets at a 100% match threshold, while a 95% threshold increased sensitivity with limited false positives. In FCoV samples, strong positives were observed with both FCoV-specific and pan-coronavirus probes, and spurious matches were negligible relative to true-positive read counts. In HIV data, specificity was preserved in controls; under the most permissive screening criterion, HIV probe matches were detected in 170/263 datasets. Subtyping using subtype-specific probes was feasible in 80 datasets, with subtype D most frequent. In colorectal cancer datasets, ISSM supported preliminary KRAS-mutation-associated probe screening. Operationally, ISSM supports rapid post-sequencing triage, prioritization of confirmatory alignments, and rational reflex testing to quantitative polymerase chain reaction/targeted sequencing, improving allocation of bioinformatics resources. Together with the NGS Dataset Downloader, the ISSM provides a user-friendly graphical-user-interface-based program for researchers and clinical users with limited bioinformatics expertise. Software and a reproducible workflow are available at https://github.com/khk1329/NDD-and-ISSM.git.

## Highlights

• NDD enables user-friendly downloading of NGS datasets from the SRA and ENA.
• The ISSM screens raw FASTQ/FASTA files for multiple probe sets without alignment.
• Parallel processing in the ISSM scales to large datasets with limited hardware.
• The sequential NDD–ISSM workflow shortens time from dataset selection to diagnostic screening.

## Graphical Abstract

**Figure:**
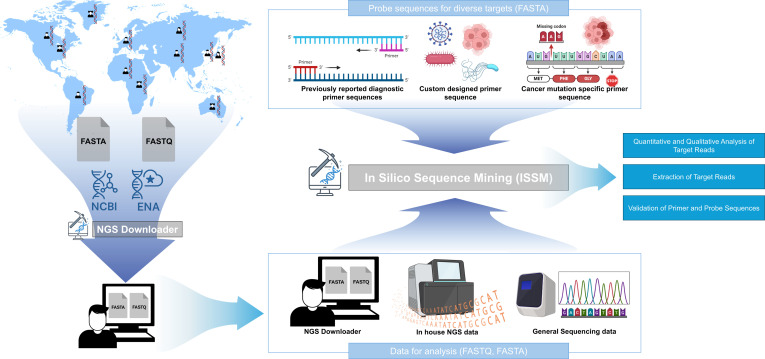

## Introduction

Since the introduction of Sanger sequencing, subsequent advances in next-generation sequencing (NGS) have transformed high-throughput sequencing across various scientific fields [1–7]. More recently, third-generation platforms such as nanopore sequencing have further expanded sequencing accessibility and portability [8]. Together, these developments have generated vast amounts of nucleotide sequence data from diverse biological and environmental samples, supporting progress in bioinformatics and advancing applications in metagenomics, transcriptomics, and public health surveillance [9–13].

Beyond basic research, NGS has become increasingly important in clinical diagnostics as automation, cost efficiency, and analytical tools have improved [14]. Targeted enrichment approaches have broadened its clinical applicability [15,16], including pathogen detection and antimicrobial resistance screening [17–19], as well as circulating tumor DNA analysis for cancer research and precision medicine [20]. As these technologies continue to evolve, high-throughput sequencing of clinical samples is becoming more widely accessible.

Accurate interpretation of large-scale NGS datasets is essential for identifying infectious agents and cancer-related genetic alterations. Several bioinformatics tools have been developed for complementary purposes, including Kraken and Kaiju for taxonomic classification [21–23] and Aperture, FACTERA, and PACT for cancer-related variant analysis [24–26]. In routine clinical or laboratory settings, however, rapid screening of predefined probe sequences may be more practical than immediate full-scale bioinformatics analysis. Tools such as BLAST and Microbe Finder can support sequence-based queries [27,28], but they are not specifically designed as lightweight graphical workflows for rapid screening of raw FASTQ/FASTA files using short user-defined probe sequences.

In parallel with the need for rapid screening, many investigators also face practical difficulties in retrieving datasets from public repositories such as the Sequence Read Archive (SRA) and the European Nucleotide Archive (ENA), particularly on Windows-based systems. To address this step, we developed the NGS Dataset Downloader (NDD), which retrieves accessions, prepares FASTQ files, and performs basic verification of the output. By delivering ready-to-screen FASTQ files with minimal setup, NDD streamlines the workflow from dataset selection to In Silico Sequence Mining (ISSM)-based analysis.

In practical workflows, investigators first curate datasets appropriate to their organism and study context, after which the ISSM provides a rapid pre-alignment screening step. Following retrieval and conversion with NDD, ISSM applies a fuzzy-match score to both forward- and reverse-complement reads and reports raw counts and percentages for each user-defined probe sequence. This supports panel sanity checks, rapid triage of related datasets, and early quality control/contamination flags without building BLAST databases or reformatting inputs. Because ISSM does not provide base-level mapping coordinates or genomic context, positive findings should be interpreted as preliminary screening results and confirmed in shortlisted candidates using alignment-based or variant-based downstream analyses.

In many clinical settings, polymerase chain reaction (PCR)-based diagnostic methods remain the standard, as the complexity of end-to-end NGS pipelines can limit accessibility. A simple and rapid method that leverages existing published primer- and probe-derived sequences while prioritizing user-friendliness could therefore improve practical applicability. Motivated by these practical workflows, we developed the graphical user interface (GUI) tool ISSM. The primary contribution of the ISSM lies in providing an accessible GUI-based workflow for rapid probe-based screening of raw sequencing datasets, rather than in the general concept of short-sequence matching itself. To support this workflow, the ISSM incorporates RapidFuzz, a text-based fuzzy string matching algorithm [29], to enable flexible approximate matching of short user-defined probe sequences in raw sequencing datasets. Rather than replacing confirmatory downstream analyses, ISSM is intended to facilitate initial screening and prioritization of candidate datasets.

## Materials and Methods

### The NDD: overview and interface

The NDD developed in this study is a user-friendly software tool designed to streamline the retrieval of publicly available NGS data. NDD accesses the SRA from the National Center for Biotechnology Information (NCBI) and the ENA as its primary data sources and enables users to retrieve datasets in FASTQ or FASTA format.

The GUI, implemented in Python using the PySide6 (Qt) framework, was designed for Windows-based environments.

The interface allows users to select a database, submit queries, manage download lists, and monitor search and download progress in real time. Search and download lists are reset when switching between databases to avoid inconsistencies, and displayed metadata are updated according to the selected source.

**Fig. 1.**
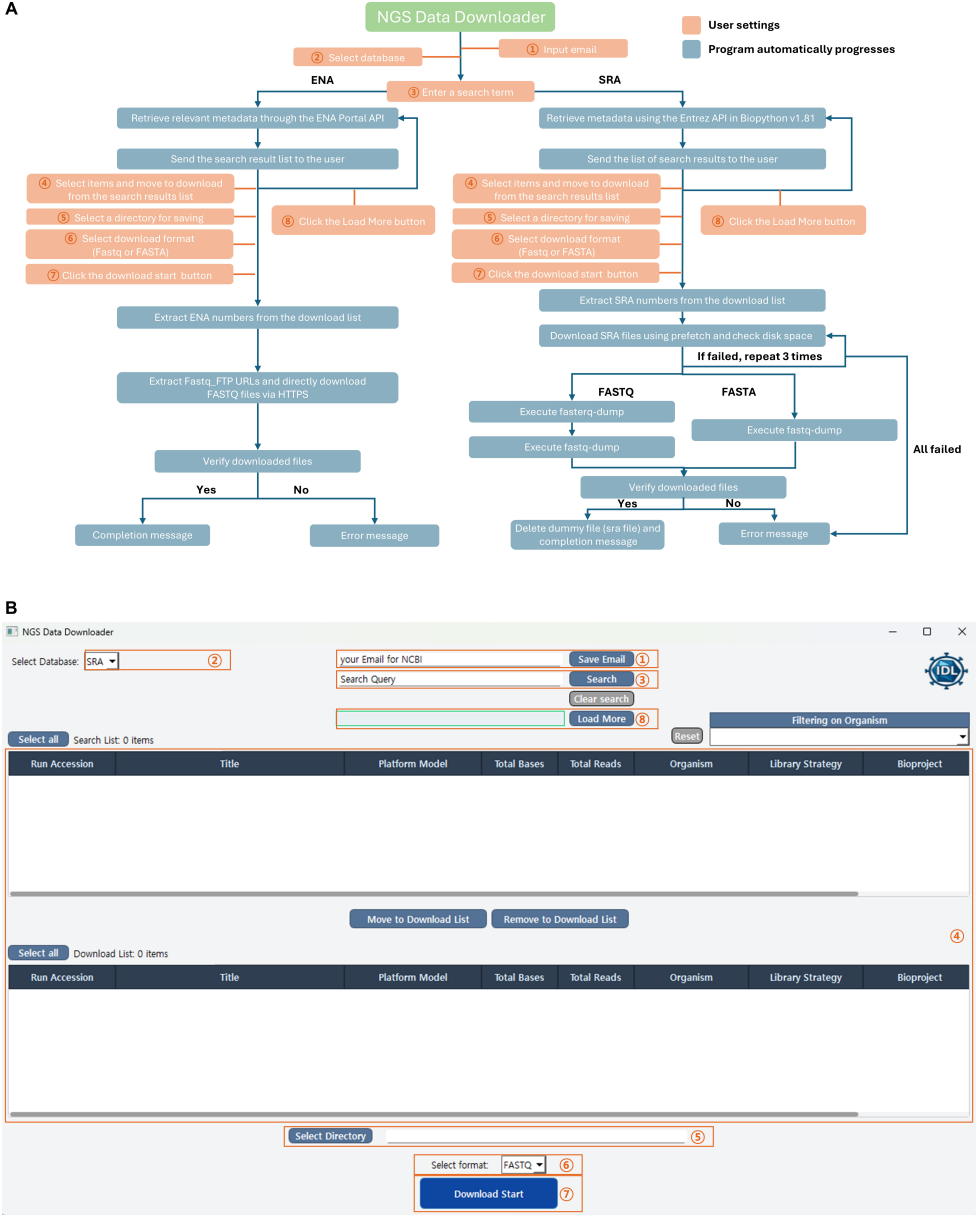
Schematic diagram and graphical user interface (GUI) of the Next-Generation Sequencing (NGS) Dataset Downloader (NDD). (A) Schematic diagram of the NDD. Orange marks components that require user input, and blue marks processes handled automatically by the software. (B) GUI of the NDD. The numbered steps in the GUI correspond to the same numbered elements in the schematic. API, application programming interface; SRA, Sequence Read Archive; ENA, European Nucleotide Archive.

User email information required for SRA access is stored in a JSON-based configuration file (config.json), which is automatically loaded at program startup for convenience (Fig. 1).

### NDD search, retrieval, and file preparation

The NDD provides an in-app search interface for both SRA and ENA. In addition to accession-based retrieval, NDD supports keyword-based searches for both repositories. Rather than enforcing a single abstracted query syntax, the current implementation handles repository-specific query semantics and result structures so that the search workflow in the GUI reflects the practical behavior users encounter on the corresponding repository websites. After a query is submitted, the application aggregates the organism field from returned records and exposes it as a selectable filter, allowing users to refine results without rerunning the search. SRA results are paged with a load-more function, whereas ENA returns the full result set in a single request. Users can further improve search precision by adding organism constraints directly to the query string. Selected entries are then transferred to the download queue for retrieval.

For SRA retrieval, metadata are obtained through the Entrez Programming Utilities (E-Utilities) using BioPython v1.81. NDD downloads run files using the official NCBI SRA Toolkit and converts them to FASTQ or FASTA format depending on the selected output option. FASTQ output is subsequently compressed with gzip to generate .fastq.gz files. If conversion with fasterq-dump fails, the program switches to fastq-dump within a bounded retry limit. For paired-end datasets, both forward and reverse read files are generated.

For ENA retrieval, the NDD queries the ENA Portal application programming interface using the user-provided search term, extracts the fastq_ftp links associated with the returned Run Accession IDs, and downloads the corresponding FASTQ files via HTTPS.

During retrieval, the NDD performs bounded automatic retries and file integrity checks and records metadata for downloaded files in CSV format. By combining search, download, file preparation, and verification within a single graphical workflow, the NDD reduces the technical burden associated with public NGS dataset acquisition (Fig. 1).

The NDD and ISSM are provided as separate GUI-based applications accessed through a common launcher. In practical use, datasets retrieved with the NDD can subsequently be analyzed in the ISSM as part of a sequential workflow, although the 2 tools do not currently operate within a single unified GUI.

### The ISSM: overview and interface

To improve the accessibility and usability of sequence mining tasks, the ISSM was developed as a GUI application for Windows-based environments, implemented in Python using the PySide6 (Qt) framework. The interface enables users to select FASTA or FASTQ input format, specify an input directory, choose files for analysis, configure analysis parameters, monitor progress in real time, and review output in a graphical environment.

The GUI is divided into a left panel and a right panel. The left panel includes settings for selecting FASTA/FASTQ formats, specifying the input folder path, and selecting files to be analyzed. The right panel consists of fields for entering probe sequences, setting the sampling ratio and matching threshold, specifying the number of files for concurrent analysis, defining the output file path, and initiating the analysis with a Start button.

**Fig. 2.**
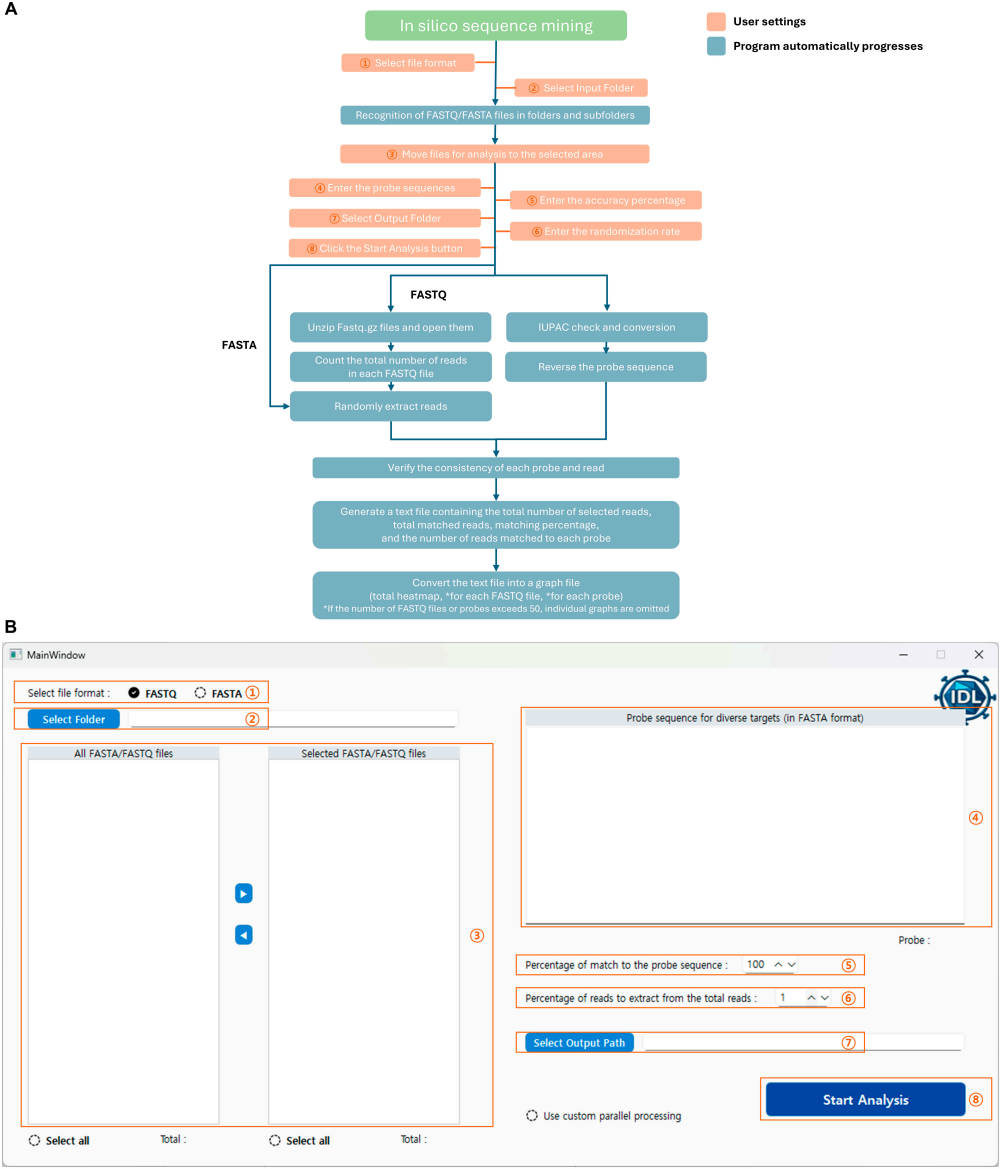
Schematic diagram and GUI of the In Silico Sequence Mining (ISSM). (A) Schematic diagram of the ISSM. Orange indicates components requiring user input and blue indicates processes handled automatically by the software. (B) GUI of the ISSM. Numbered steps in the GUI correspond to identically numbered elements in the schematic.

Communication between the GUI and the backend is facilitated by Qt components, such as QTimer, QProgressBar, QLabel, and QDialog, and is updated in real time using Python’s threading module and queue.Queue (Fig. 2).

### Preparing analysis data and probe sequences

The data used for analysis consisted of FASTA or FASTQ files obtained from NGS experiments or public repositories such as the SRA and ENA. The ISSM supports both compressed (.fastq.gz, .fq.gz, .fasta.gz, and .fa.gz) and uncompressed (.fastq, .fq, .fasta, .fa, and .fna) file formats. Users can select the desired folder through the GUI, and the program recursively scans the folder and its subdirectories to identify and display files matching the selected input format. From this list, users can select the files to be included in the analysis.

Probe sequences are entered by the user through the GUI in FASTA format, including the standard header line beginning with “>”. Before analysis, the probe input is validated to confirm proper FASTA structure, the presence of non-empty probe names and sequences, the absence of duplicate probe names, and the use of valid nucleotide characters only.

The ISSM supports degenerate nucleotide sequences using International Union of Pure and Applied Chemistry codes in the probe input. During preprocessing, ambiguous bases are expanded into all possible sequence combinations and cached for downstream matching. No fixed hard limit is imposed on probe length or probe number at the interface level. However, the computational burden increases as probe number, probe length, and the degree of sequence degeneracy increase because ambiguous International Union of Pure and Applied Chemistry codes are expanded into all possible sequence combinations during preprocessing (Fig. 2).

### Sampling strategy

To efficiently process large-scale sequencing datasets, a random sampling strategy was implemented to select a user-defined percentage of input sequences. The sampling ratio can be set as an integer between 1% and 100%, with a minimum sample size of 5,000 reads enforced for FASTQ files. For FASTA files, all records are first parsed into a list, after which a random subset is selected using Python’s random.sample function.

Because FASTQ files are typically larger and more memory intensive, a double-pass sampling approach was employed to improve memory efficiency and processing speed. In the first pass, the total number of reads is counted, and the desired number of samples is calculated. A set of random indices corresponding to the selected reads is then generated. In the second pass, only the reads matching these indices are extracted. The sampled FASTQ reads are saved as temporary compressed files using the gzip module for downstream analysis (Fig. 2).

### Sequence matching and parallel processing

The input sequencing reads are matched against the user-provided probe sequences based on substring similarity. The similarity comparison is implemented using the fuzz.partial_ratio() function from the RapidFuzz library, which provides a high-speed approximate matching method based on Levenshtein distance. In this framework, substitutions, insertions, and deletions reduce the similarity score reported by fuzz.partial_ratio(), and the user-defined threshold determines whether a read is retained as a preliminary match. Each read is compared with both its original nucleotide sequence and its reverse complement. If either version yields a similarity score equal to or above the user-defined threshold, it is considered a match. Reads shorter than the corresponding probe sequence are excluded from matching.

To improve computational efficiency, parallel processing is employed. Each worker process preloads the shared probe sequences via an initializer function, reducing redundant data copying and optimizing memory usage. The number of concurrent processes is automatically adjusted based on the user’s central processing unit (CPU) core count, with an option for manual configuration.

The analysis method differs depending on the input file format. For FASTA files, sequences are loaded into memory using Bio.SeqIO.parse() and processed in batches in parallel using Python’s concurrent.futures.ProcessPoolExecutor. Each batch is compared against the shared set of probe sequences.

For FASTQ files, which typically contain larger volumes of data, a hybrid approach combining parallel and batch-based processing is used to enhance performance and memory stability. The FASTQ files analyzed are the temporary compressed files generated during the sampling step. These files are processed using gzip.open() for streaming text access. Reads are divided into batches of 500, and each batch is analyzed in parallel using ProcessPoolExecutor. This design reduces interprocess communication overhead and mitigates input/output bottlenecks, resulting in improved processing speed (Fig. 2).

### Output and visualization

Upon completion of the analysis, the results for each input file are saved in tab-delimited .txt files. The output includes the total number of selected reads or sequences, the number of matched reads or sequences for each probe, and detailed per-read/per-sequence matching information, including the probe name and similarity scores. By default, 3 main types of text output files are generated. These include file-specific result files (<filename>_results.txt), a summary across all input files (summary_result_<YYYYMMDD_HHMMSS>.txt), and an aggregated result file (total_results.txt). For each input file, matched reads or sequences are also saved as separate FASTA files (<filename>_matched_reads.fasta) for downstream validation.

Visualization is performed automatically using the matplotlib and seaborn libraries. The visual outputs include bar graphs and heatmaps, which facilitate intuitive interpretation of probe-sequence matching patterns. These saved FASTA outputs can be used for secondary alignment, locus verification, or phylogenetic analysis, offering a flexible follow-up to initial probe-based screening.

Two types of bar graphs are generated. One displays the match count per probe for each individual file, while the other enables cross-file comparison of match frequencies per probe. These visualizations are implemented using the matplotlib.pyplot.bar() function. File-specific bar plots are stored in the input_files/ subdirectory, whereas probe-specific bar plots are stored in the probes/ subdirectory.

For the heatmap, the results are represented as a 2-dimensional matrix, with probe names as rows, file names as columns, and matching percentages as values. The total_results.txt file is loaded using pandas.read_csv(), and a DataFrame is constructed using pivot(). The heatmap is visualized using seaborn.heatmap() with the coolwarm colormap, providing an intuitive representation of relative matching intensities.

All visualizations are saved in .png format at 300-dpi resolution. The heatmap image (Total_heatmap.png) and corresponding bar graphs are stored within the analysis output directory, with the heatmap saved in the main output folder and the bar graphs in the respective “input_files/” and “probes/” subdirectories (Fig. 2).

### Evaluation and run reporting

For the NDD, retrieval jobs were evaluated based on whether the expected output files were successfully generated and accessible after download and file preparation. The software recorded job completion status and elapsed time within the session.

For the ISSM, each analysis run generated per-file probe-matching summaries together with total selected read/sequence counts, matched read/sequence counts, and elapsed time. These run summaries were used for performance tracking and for comparison across datasets analyzed under identical parameter settings, thereby supporting side-by-side evaluation under consistent analysis conditions.

### NDD performance evaluation

To evaluate NDD performance, we used the PRJNA898830 NGS dataset and downloaded 263 FASTQ files across 2 computing environments. File sizes ranged from approximately 0.08 to 1,065.26 MB, and per-file read counts were roughly between 649 and 7,506,398 (Table S1).

Two computing environments were used for the evaluation, comprising a personal computer (PC) and a workstation (WS). The PC was equipped with an Intel Core i5-14500 CPU, 64 GB random access memory (RAM), and ran Windows 11 Home (version 24H2). The WS was configured with an Intel Xeon Gold 6230R CPU, 256 GB RAM, and operated on Windows 10 Pro (version 2009).

Storage paths were configured on both solid state drive (SSD) and hard disk drive (HDD) in each system. On the PC, the SSD was SK hynix SHGP31 500GM and the HDD was Seagate ST1000DM014 2UB10D. On the WS, the SSD was SK hynix PC801 NVMe 1 TB and the HDD was DELL PERC H330 Adp SCSI Disk Device. In addition, the external HDD commonly used across both systems was a Toshiba MQ01ABD100, connected via a JMicron Tech SCSI Disk Device.

### Scalability and runtime evaluation of the ISSM using parallel file processing

To evaluate the computational efficiency of the ISSM, 12 FASTQ files were analyzed under consistent conditions on 2 systems with different hardware specifications. The FASTQ files ranged in size from 82 to 528 MB, containing between 1,005,781 and 6,342,416 reads.

Two computing environments were used for the evaluation, PC and WS. The directory containing the FASTQ files to be analyzed and the directory for saving the analysis results were located on the SSD of both the PC and the WS.

The ISSM option “Use custom parallel processing” was enabled during the tests. Performance was assessed by varying the number of files processed concurrently to 4, 8, and 12. Primer sequences targeting HIV were used in these tests (Table 1) [30–33].

**Table 1.** Probe sequences applied in the analysis of HIV-related next-generation sequencing (NGS) datasets using In Silico Sequence Mining (ISSM)

No.	Probe name	Target HIV-1 subtype	Sequence	Reference
1	BECO5	Universal	GGCATCAAACAGCTCCAGGCAAG	[30]
2	BECO3		AGCAAAGCCCTTTCTAAGCCCTGTCT	
3	BE-ANCH		TCCTGGCTGTGGAAAGATACCTA	
4	B-SPEC	B and D	GTCCCCTCGGGGCTGGGAGG	
5	E-SPEC	A, E, and G	GTCTCAGTCCCTTGAGACTGCTG	
6	F-SPEC	F	AACAGCTCTACCAGCTCTTTGCAAA	
7	C-SPEC	C	AGACCCCAATACTGCACAAGACTT	
8	5′E	E	CAGGAAAGGAATGAAAAGGATTTGTTA	
9	3′E		ATAACCCTATCTGTCCACCCC	
10	5′A	A	GANAACATGACCTGGCTGC	
11	3′A		TCTATAACCCTATCTGTCCAGCCA	
12	5′G	G	ACAATTACACATACCACATATACAGCC	
13	3′G		TCTATAACCCTATCTGTCCAGTT	
14	5′D	D	ACCACTAATGTGCCCTGGAACT	
15	3′D		AGGAGGGTCTGAAATGACAGA	
16	SK29	Universal	ACTAGGGAACCCACTGCT	[31]
17	SK30		GGTCTGAGGGATCTCTA	
18	SK31		ACCAGAGTCACACAACAGACGGGCACACACTACT	
19	SK38		ATAATCCACCTATCCCAGTAGGAGAAAT	
20	SK39		TTTGGTCCTTGTCTTATGTCCAGAATGC	
21	SK19		ATCCTGGGATTAAATAAAATAGTAAGAATGTATAGCCCTAC	
22	SK68		AGCAGCAGGAAGCACTATGG	
23	SK69		CCAGACTGTGAGTTGCAACAG	
24	SK70		ACGGTACAGGCCAGACAATTATTGTCTGGTATAGT	
25	CO1		ACAATTATTGTCTGGTATAG	
26	CO2		AGGTATCTTTCCACAGCCAG	
27	CO3		TGAGTTGCAACAGATGCTGTTGCGCCTCAATAGCCCTCAG	
28	JA167		TATCYTTTGAGCCAATTCCYATACA	[32]
29	JA168		ACAATGYACACATGGAATTARGCCA	
30	JA169		AGAAAAATTCYCCTCYACAATTAAA	
31	JA170		GTGATGTATTRCARTAGAAAAATTC	
32	Beta-actin-F	–	AGAGATGGCCACGGCTGCTT	[33]
33	Beta-actin-R		ATTTGCGGTGGACGATGGAG	

### ISSM analysis of a FASTA file containing diverse coronavirus genomic sequences using published coronavirus-targeting primer- and probe-derived sequences

In this study, we evaluated how well published PCR and quantitative PCR primer- and probe-derived sequences for coronavirus detection function as probe inputs in the ISSM for identifying coronavirus genomic sequences. To perform the analysis, coronavirus-related entries were retrieved from the NCBI Nucleotide Database using the following filtering criteria: species set to “Viruses,” molecule type set to “Genomic DNA/RNA,” and sequence length set to ≥25,000 bp. The resulting sequences were then used in the analysis.

From the search results, FASTA files corresponding to each coronavirus type were downloaded. The downloaded datasets included Alphacoronavirus 1 (*n* = 255), Human coronavirus 229E (*n* = 318), Human coronavirus NL63 (*n* = 327), Human coronavirus HKU1 (*n* = 329), Middle East respiratory syndrome-related coronavirus (*n* = 809), Avian coronavirus (*n* = 817), Betacoronavirus 1 (*n* = 1,014), Porcine epidemic diarrhea virus (*n* = 1,030), and SARS-CoV-2 (*n* = 30,000). Sequences for each type were concatenated into a single FASTA file for analysis.

The probe dataset included sequences derived from 16 universal coronavirus primers, 9 SARS-CoV primers, 43 SARS-CoV-2 primers, 2 bovine coronavirus primers, 20 Alphacoronavirus primers, 3 feline coronavirus primers, 19 primers targeting other viruses, 5 CRFK cell sequences, and 2 beta-actin sequences (Table 2) [33–51].

**Table 2.** Probe sequences applied in the analysis of coronavirus-related NGS datasets using the ISSM

No.	Target	Target gene	Probe name	Sequence (5′→3′)	Reference
1	SARS-CoV-2	ORF-1ab	CDC-F	CCCTGTGGGTTTTACACTTAA	[34]
2			CDC-p	CCGTCTGCGGTATGTGGAAAGGTTATGG	
3			CDC-R	ACGATTGTGCATCAGCTGA	
4		N	CDC-N-F	GGGGAACTTCTCCTGCTAGAAT	
5			CDC-N-P	TTGCTGCTGCTTGACAGATT	
6			CDC-N-R	CAGACATTTTGCTCTCAAGCTG	
7		ORF-1b	HKU-ORF1b-F	TGGGGYTTTACRGGTAACCT	[35]
8			HKU-ORF1b-P	TAGTTGTGATGCWATCATGACTAG	
9			HKU-ORF1b-R	AACRCGCTTAACAAAGCACTC	
10		N	HKU-N-F	TAATCAGACAAGGAACTGATTA	
11			HKU-N-P	GCAAATTGTGCAATTTGCGG	
12			HKU-N-R	CGAAGGTGTGACTTCCATG	
13		N	USA-F-1	GACCCCAAAATCAGCGAAAT	[36]
14			USA-P-1	ACCCCGCATTACGTTTGGTGGACC	
15			USA-R-1	TCTGGTTACTGCCAGTTGAATCTG	
16		N	USA-F-2	TTACAAACATTGGCCGCAAA	
17			USA-P-2	ACAATTTGCCCCCAGCGCTTCAG	
18			USA-R-2	GCGCGACATTCCGAAGAA	
19		N	USA-F-3	GGGAGCCTTGAATACACCAAAA	
20			USA-P-3	AYCACATTGGCACCCGCAATCCTG	
21			USA-R-3	TGTAGCACGATTGCAGCATTG	
22		RdRp	GRT-F	GTGARATGGTCATGTGTGGCGG	[37]
23			GRT-P1	CAGGTGGAACCTCATCAGGAGATGC	
24			GRT-P2	CCAGGTGGWACRTCATCMGGTGATGC	
25			GRT-R	CARATGTTAAASACACTATTAGCATA	
26		E	GRT-E-F	ACAGGTACGTTAATAGTTAATAGCGT	
27			GRT-E-P	ACACTAGCCATCCTTACTGCGCTTCG	
28			GRT-E-R	ATATTGCAGCAGTACGCACACA	
29		N	GRT-N-F	CACATTGGCACCCGCAATC	
30			GRT-N-P	ACTTCCTCAAGGAACAACATTGCCA	
31			GRT-N-R	GAGGAACGAGAAGAGGCTTG	
32		RdRp	Paris-IP2-F	ATGAGCTTAGTCCTGTTG	[38]
33			Paris-IP2-R	CTCCCTTTGTTGTGTTGT	
34			Paris-IP2-P	AGATGTCTTGTGCTGCCGGTA	
35		RdRp	Paris-IP4-F	GGTAACTGGTATGATTTCG	
36			Paris-IP4-R	CTGGTCAAGGTTAATATAGG	
37			Paris-IP4-P	TCATACAAACCACGCCAGG	
38		N	Jap-F	AAATTTTGGGGACCAGGAAC	[39]
39			Jap-R	TGGCAGCTGTGTAGGTCAAC	
40			Jap-P	ATGTCGCGCATTGGCATGGA	
41		N	Thailand-F	CGTTTGGTGGACCCTCAGAT	[40]
42			Thailand-R	CCCCACTGCGTTCTCCATT	
43			Thailand-P	CAACTGGCAGTAACCA	
44	SARS-CoV	ORF-1ab	Pan-SARS-ORF1ab-F	GGAAAGGTTATGGCTGTAGTTGTG	[34]
45			Pan-SARS-ORF1ab-R	CCGCACGGTGTAAGACGG	
46			Pan-SARS-ORF1ab-P	AACGGGTTTGCGGTGTAAGTGCAG	
47		E	Pan-SARS-E-F	ACACTAGCCATCCTTACTG	
48			Pan-SARS-E-R	CACGTTAACAATATTGCAGC	
49			Pan-SARS-E-P	CGCTTCGATTGTGTGCGTACT	
50		N	Pan-SARS-N-F	ACATTGGCACCCGCAATCC	
51			Pan-SARS-N-R	GCTTGACTGCCGCCTCTGCT	
52			Pan-SARS-N-P	CGTGCTACAACTTCCTCAAGGAACA	
53	Bovine CoV	N	BCoV_Forward	CTAGTAACCAGGCTGATGTCAATACC	[41]
54			BCoV_Reverse	GGCGGAAACCTAGTCGGAATA	
55	Alphacoronavirus	ORF 1a	AC01-F	TTTGTGTCTACTYYTCTCAACTA	[42]
56			AC01-R	GCATTMACCCAACAATTATTRT	
57		ORF 1a	AC02-F	TTTAATGTTGTWGGKCCICG	
58			AC02-R	GCATAACAAGCRCARCGRTA	
59		ORF 1a	AC03-F	GGTGGTRAYAATGTTTATTGYTA	
60			AC03-R	AGRCCAAAATCACTRTGYTTA	
61		ORF 1a	AC04-F	TAYCGYTGYGCTTGTTATGC	
62			AC04-R	GGWACACCATCWATAAAMAC	
63		ORF 1ab	AC05-F	CTGGTARYGGTCARGCTAT	
64			AC05-R	CAYTTAGTRCACAACATMGG	
65		ORF 1ab	AC06-F	GTKTTTATWGATGGTGTWCC	
66			AC06-R	ACATCCATWCCYAACCAACC	
67		ORF 1b	AC07-F	CCKATGTTGTGYACTAARTG	
68			AC07-R	CTRCCATCRTACATATCWGA	
69		ORF1b~S	AC08-F	GGTTGGTTRGGWATGGATGT	
70			AC08-R	TAAGGCRTCTTCWATRGTTTTACA	
71		S	AC09-F	TATGGTGATGTKTCWAAAACTAC	
72			AC09-R	TCRTAATAAGGAAGTTTAGTTGA	
73		S~All	AC10-F	TGTAAAACYATWGAAGAYGCCTTA	
74			AC10-R	AAAAATGGCTCTTCCATTGTTGGC	
75	Universal coronavirus 1	RdRp	Chu-RdRp-N1-F	GGKTGGGAYTAYCCKAARTG	[43]
76			Chu-RdRp-N1-R	TGYTGTSWRCARAAYTCRTG	
77			Chu-RdRp-N2-F	GGTTGGGACTATCCTAAGTGTGA	
78			Chu-RdRp-N2-R	CCATCATCAGATAGAATCATCAT	
79	Universal coronavirus 2	RdRp	Bat-Cov-RdRp-1-F	AYAACCAAGATCTTAATGG	[44]
80			Bat-Cov-RdRp-1-R	TGCTTAGAACCCAAAATCAT	
81			Bat-Cov-RdRp-2-F	GGTTGGGACTATCCTAAGTGTGA	
82			Bat-Cov-RdRp-2-R	CCATCATCAGATAGAATCATCATA	
83		Helicase-ExoN	Bat-Cov-Heli-F	CTCARGGTAGTGARTATGA	
84			Bat-Cov-Heli-R	AATTGTTCWCCWGGTGG	
85		Spike	Bat-Cov-S-1-F	WTATGTTTGYAATGGTAAY	
86			Bat-Cov-S-1-R	GTCWTCATCMACWGTRC	
87			Bat-Cov-S-2-F	GAYTDDCAGCACTTAATGC	
88			Bat-Cov-S-2-R	TTGAGCCAYTCAAGRTYRA	
89			Bat-Cov-S-3-F	CAATCTAGGTCTGCTATCG	
90			Bat-Cov-S-3-R	CTAGAAGACTGTGATTTGA	
91	Feline coronavirus	ORF1	FcovP	TCCATTGTTGGCTCGTCATAGCGGA	[45]
92			FcovF	GATTTGATTTGGCAATGCTAGATTT	
93			FcovR	AACAATCACTAGATCCAGACGTTAGCT	
94	Feline parvovirus	ORF1	FPVp	CTGGGGGTGTGGGGATTTCTACG	[46]
95			FPVf	CGGGGGTGGTGGTGGTT	
96			FPVr	GCTTGAGTTTGCTGTGATTTCC	
97	Hantavirus	L	HNL-2111F	CARTCWACWGTGGGCAGTGG	[47]
98			HANL-R1	AACCADTCWGTYCCRTCATC	
99			HANL-R2	GCRTCRTCWGARTGRTGDGCAA	
100		S	HHSIF	GTAAGCCCTGTCATGAGTGTAG	[48]
101			HHSIQ	TTGCATGTTGCCTGAGGGCTTGA	
102	Paramyxovirus	RdRp	PAR-F1	GAAGGTATTGTCAAARNTNTGGAC	[49]
103			PAR-F2	GTTGCTTCAATGGTTCARGGNGAYAA	
104			PAR-R	GCTGAAGTTACGGTCCCDATRTTNC	
105	Papillomavirus	L1	BconPVF	TYCCWAAGGTSTCTGSAAATCA	[50]
106			BconPVR	GTACGACTCACTATAGGGA	
107	Influenza virus	M	M30F2/08	ATGAGYCTTYTAACCGAGGTCGAAACG	[51]
108			M264R3/08	TGGACAAANCGTCTACGCTGCAG	
109		HA	H5-918F	CCARTRGGKGCKATAAAYTC	
110			H5-671-647R	CTCTGGTTTAGTGTTGATGTYCCAA	
111			H1-HARend	AGTAGAAACAAGGGTGTTTTT	
112			H1-HAFA	GGGAGAATGAACTATTACTGG	
113	CRFK cell	CRFK cell	OR237969.1 CRFK_cell_DDX20_1	TACCATTGCTTTGGACTCTC	-
114			OR237969.1 CRFK_cell_DDX20_2	CTGGCCGTTTTGGTACCTTG	
115			OR237969.1 CRFK_cell_DDX20_3	CCTGTGAAAAGCCACTCAGA	
116			OR237969.1 CRFK_cell_DDX20_4	CCTGAAGAGAGAGTGCAGGT	
117			OR237969.1 CRFK_cell_DDX20_5	GGACGGTTGGTACGACTGCC	
118	Human beta-actin	Beta-actin	Beta-actin-F	AGAGATGGCCACGGCTGCTT	[33]
119			Beta-actin-R	ATTTGCGGTGGACGATGGAG	

During the analysis, the program settings were configured as follows: the “Percentage of match to the probe sequence” was set to 100%, and the “Percentage of reads to extract from the total reads” was also set to 100%.

### Performance validation of the ISSM using coronavirus FASTQ datasets generated in CRFK cells

CRFK cells provided by the Korea Research Institute of Bioscience and Biotechnology were cultured in Eagle’s minimum essential medium (EMEM) supplemented with 10% fetal bovine serum (FBS; Gibco, USA) and 1× antibiotic-antimycotic (100 U/ml penicillin, 100 μg/ml streptomycin, and 0.25 μg/ml amphotericin B; Gibco, USA) at 37 °C in a 5% CO_2_ incubator and subsequently used for virus infection. The feline coronavirus (FCoV) type 2 strain WSU 79-1683 (American Type Culture Collection VR-989) was obtained from the American Type Culture Collection and used in this study. CRFK cells (1 × 10^6^/ml) were seeded in 25-cm^2^ tissue culture flasks and cultured as monolayers in EMEM (10% FBS). The FCoV was diluted 1:10 in EMEM and incubated for 4 d. Cytopathic effects were observed under an inverted light microscope, and the 50% tissue culture infectious dose (TCID_50_) of the passage 10 (p10) FCoV was determined to be 10^4.7^ TCID_50_/ml.

For performance evaluation, CRFK cells were seeded at a density of 8 × 10^5^/ml in 25-cm^2^ flasks and incubated overnight. Virus infection was performed in 2 rounds. After the overnight culture, cells were infected with p10 FCoV at a multiplicity of infection of 0.001 in EMEM (10% FBS). After 24 h, a second infection was carried out under the same conditions, while uninfected control flasks were maintained in EMEM (10% FBS) for parallel culturing. For cell-culture-supernatant samples, medium from uninfected flasks was harvested and processed in the same manner as infected cultures.

All flasks were cultured for 4 d postinfection and harvested. Cells were passaged up to passage 3 (P3), while nonpassaged cells served as the negative control. Culture supernatants from passages 1 and 3 were collected and subjected to RNA extraction.

RNA extraction was performed using TRIzol LS (Ambion, USA) following the manufacturer’s protocol. The eluate was treated with 30 μl of diethyl pyrocarbonate (DyneBio, South Korea). The extracted RNA was sent to Macrogen (Seoul, South Korea) for library preparation using the TruSeq Stranded Total RNA LT Sample Prep Kit (Ribo-Zero H/M/R Gold), followed by Illumina NovaSeq 6000 sequencing (5-Gb output, paired-end 100 bp).

The resulting paired-end FASTQ reads were paired using Geneious Prime software and trimmed using the BBDuk tool to remove all Truseq, Nextera, and PhiX adapter sequences (158 total). Reads with base quality scores below 30 were filtered out. The resulting data were saved in .fastq.gz format for further analysis.

Sequence analysis was performed using the ISSM workflow implemented in the developed software. A total of 119 published primer- and probe-derived sequences, including 93 coronavirus-related sequences (Table 2) [33–51], were used.

To further quantify the reliability of downsampling-based estimation in the FCoV-related datasets, ISSM analysis was first performed at a 100-score threshold with 100% read extraction, and the matched read count obtained for each probe under this condition was defined as the reference value. The matched read counts obtained for the same probes under the 1%, 10%, 25%, and 50% extraction settings were then collected and converted into percentages relative to the corresponding 100% extraction values. Using these data, the relationship between the actual extraction fraction and the detected proportion relative to the 100% extraction value was modeled by linear regression. The resulting regression equation was subsequently used to back-calculate 100% equivalent matched read counts from the observed matched read counts obtained under each extraction setting. Relative error was then calculated from the difference between the back-calculated value and the actual matched read count obtained under 100% extraction. Finally, the relationship between the actual matched read abundance at 100% extraction and the relative error was analyzed to evaluate the reliability of downsampling-based estimation across extraction fractions.

### ISSM-based detection and subtyping of HIV-1 in clinical FASTQ datasets

This analysis aimed to evaluate how well HIV-1 primer- and probe-derived sequences function as probe inputs in the ISSM for detecting and subtyping HIV-1 in real clinical samples and whether subtype-level screening is feasible [30–33]. To this end, serum samples from patients with sepsis infected with HIV were analyzed using NGS. The NGS dataset, registered under BioProject number PRJNA898830 in the NCBI SRA database, was obtained using the NGS downloader function of the software. The dataset included 9 control group samples and a total of 263 individual sequencing files [52]. The collected datasets were generated in paired-end format and used directly for analysis without additional preprocessing. In total, 526 .fastq.gz files were included. The published HIV-1 primer- and probe-derived sequences were provided by the user in FASTA format and used as probe inputs in ISSM, and 33 probe sequences were analyzed in total, including 2 beta-actin probe sequences (Table 1).

During the analysis, the “Percentage of match to the probe sequence” option was set to 100, and the “Percentage of reads to extract from the total reads” option was set to 1, 3, 5, 10, and 100. For conditions in which the sampling rate was less than 100%, each condition was analyzed in triplicate to ensure reproducibility.

To examine the effect of probe length on matched read counts, SK30, which showed matched reads in the largest number of datasets, was used as the reference probe. The region corresponding to SK30 was identified in the NCBI HIV-1 reference sequence (NC_001802), and a probe set comprising 101 sequences ranging from 5 to 105 bp in 1-bp increments was constructed (Table 3). The probe set was then applied to 6 paired datasets (SRR22211849, SRR22211876, SRR22211877, SRR22211898, SRR22211958, and SRR22212049) with high matched read counts for SK30, and matched read counts at each probe length were obtained. The matched read count at 5 bp was used as the reference value (100%) for relative comparison.

**Table 3.** Probe sequence set comprising 101 sequences ranging from 5 to 105 bp, derived from the HIV-1 probe sequence SK30 based on the NCBI HIV-1 reference sequence (NC_001802)

No.	Probe length	Sequence
1	5 bp	GGTCT
2	6 bp	GGTCTG
3	7 bp	GGTCTGA
4	8 bp	GGTCTGAG
5	9 bp	GGTCTGAGG
6	10 bp	GGTCTGAGGG
7	11 bp	GGTCTGAGGGA
8	12 bp	GGTCTGAGGGAT
9	13 bp	GGTCTGAGGGATC
10	14 bp	GGTCTGAGGGATCT
11	15 bp	GGTCTGAGGGATCTC
12	16 bp	GGTCTGAGGGATCTCT
13	17 bp (SK30)	GGTCTGAGGGATCTCTA
14	18 bp	GGTCTGAGGGATCTCTAG
15	19 bp	GGTCTGAGGGATCTCTAGT
16	20 bp	GGTCTGAGGGATCTCTAGTT
17	21 bp	GGTCTGAGGGATCTCTAGTTA
18	22 bp	GGTCTGAGGGATCTCTAGTTAC
19	23 bp	GGTCTGAGGGATCTCTAGTTACC
20	24 bp	GGTCTGAGGGATCTCTAGTTACCA
21	25 bp	GGTCTGAGGGATCTCTAGTTACCAG
22	26 bp	GGTCTGAGGGATCTCTAGTTACCAGA
23	27 bp	GGTCTGAGGGATCTCTAGTTACCAGAG
24	28 bp	GGTCTGAGGGATCTCTAGTTACCAGAGT
25	29 bp	GGTCTGAGGGATCTCTAGTTACCAGAGTC
26	30 bp	GGTCTGAGGGATCTCTAGTTACCAGAGTCA
27	31 bp	GGTCTGAGGGATCTCTAGTTACCAGAGTCAC
28	32 bp	GGTCTGAGGGATCTCTAGTTACCAGAGTCACA
29	33 bp	GGTCTGAGGGATCTCTAGTTACCAGAGTCACAC
30	34 bp	GGTCTGAGGGATCTCTAGTTACCAGAGTCACACA
31	35 bp	GGTCTGAGGGATCTCTAGTTACCAGAGTCACACAA
32	36 bp	GGTCTGAGGGATCTCTAGTTACCAGAGTCACACAAC
33	37 bp	GGTCTGAGGGATCTCTAGTTACCAGAGTCACACAACA
34	38 bp	GGTCTGAGGGATCTCTAGTTACCAGAGTCACACAACAG
35	39 bp	GGTCTGAGGGATCTCTAGTTACCAGAGTCACACAACAGA
36	40 bp	GGTCTGAGGGATCTCTAGTTACCAGAGTCACACAACAGAC
…	…	…
97	101 bp	GGTCTGAGGGATCTCTAGTTACCAGAGTCACACAACAGACGGGCACACACTACTTGAAGCACTCAAGGCAAGCTTTATTGAGGCTTAAGCAGTGGGTTCCC
98	102 bp	GGTCTGAGGGATCTCTAGTTACCAGAGTCACACAACAGACGGGCACACACTACTTGAAGCACTCAAGGCAAGCTTTATTGAGGCTTAAGCAGTGGGTTCCCT
99	103 bp	GGTCTGAGGGATCTCTAGTTACCAGAGTCACACAACAGACGGGCACACACTACTTGAAGCACTCAAGGCAAGCTTTATTGAGGCTTAAGCAGTGGGTTCCCTA
100	104 bp	GGTCTGAGGGATCTCTAGTTACCAGAGTCACACAACAGACGGGCACACACTACTTGAAGCACTCAAGGCAAGCTTTATTGAGGCTTAAGCAGTGGGTTCCCTAG
101	105 bp	GGTCTGAGGGATCTCTAGTTACCAGAGTCACACAACAGACGGGCACACACTACTTGAAGCACTCAAGGCAAGCTTTATTGAGGCTTAAGCAGTGGGTTCCCTAGT

### Detection of KRAS mutations in colorectal cancer NGS data using the ISSM

This study also aimed to evaluate whether the ISSM could distinguish between primary colorectal tumor tissue and adjacent normal mucosal tissue using clinical NGS datasets from patients with colorectal cancer. This KRAS-related analysis was intended as a proof-of-concept example to demonstrate the applicability of the ISSM to oncology-associated NGS datasets rather than as a comprehensive survey of all clinically relevant KRAS mutations. To this end, primer-derived sequences targeting representative KRAS mutations (G12A, G12C, G12D, G12R, G12S, G12V, and G13D) together with normal KRAS-related sequences were selected and used as probe sequences for the analysis [53]. To verify the presence of wild-type KRAS in both primary colorectal tumor tissue and adjacent normal mucosal tissue datasets, Geneious Prime software was used to perform Geneious Alignment at 65% similarity between the KRAS reference gene (NC_000012.12) and the selected primers. The binding regions of the primers were identified, and 9 probe variants (Kras-A through Kras-I) were designed accordingly for inclusion in the analysis (Table 4).

**Table 4.** Probe sequences applied in the analysis of colorectal-cancer-related NGS datasets using the ISSM

No.	Probe name	Target	Sequence	Reference
1	Kras G12A inv k	KRAS mutation	TCTTGCCTACGCCACG	[53]
2	Kras G12C inv k		TCTTGCCTACGCCACA	
3	Kras G12D inv k		CTCTTGCCTACGCCAT	
4	Kras G12R inv k		TCTTGCCTACGCCACG	
5	Kras G12S inv k		TCTTGCCTACGCCACT	
6	Kras G12V inv k		CTCTTGCCTACGCCAA	
7	Kras G13D inv k		GCACTCTTGCCTACGT	
8	Kras A1 inv k	KRAS	GCCTGCTGAAAATGACTGA	
9	Kras B1 inv k		CCTTGGGTTTCAAGTTATATG	
10	Kras B2 inv k		CCCTGACATACTCCCAAGGA	
11	Kras-A		CATCTCTTGCCCTCTCAAAG	This study
12	Kras-B		CTCTTGCCCTCTCAAAGTGC	
13	Kras-C		GCCCTCTCAAAGTGCTGGT	
14	Kras-D		TGGCATCTCTTGCC	
15	Kras-E		CATCTCTTGCCCTCTCAAAG	
16	Kras-F		GCCCTCTCAAAGTGCTGGT	
17	Kras-G		CTCTCAAAGTGCTG	
18	Kras-H		CAAAGTGCTGGTAC	
19	Kras-I		GTGCTGGTACTACA	
20	Beta-actin-F	Human beta-actin	AGAGATGGCCACGGCTGCTT	[33]
21	Beta-actin-R		ATTTGCGGTGGACGATGGAG	

The dataset consisted of samples from 60 colorectal cancer patients, including paired NGS datasets from primary colorectal tumor tissue and adjacent normal mucosal tissue. These datasets were obtained from the NCBI SRA (BioProject PRJEB1752) using the NGS downloader function, yielding a total of 120 NGS datasets (60 tumor and 60 normal) [54]. All files were generated in paired-end format and were analyzed in their original state without any preprocessing. A total of 240 .fastq.gz files were included in the analysis. The selected sequences were provided in FASTA format and used as probe inputs in the ISSM.

During the ISSM analysis, both the “Percentage of match to the probe sequence” and the “Percentage of reads to extract from the total reads” options were set to 100%, enabling full-scale evaluation of probe matching across all reads.

## Results

### NDD performance evaluation

**Fig. 3.**
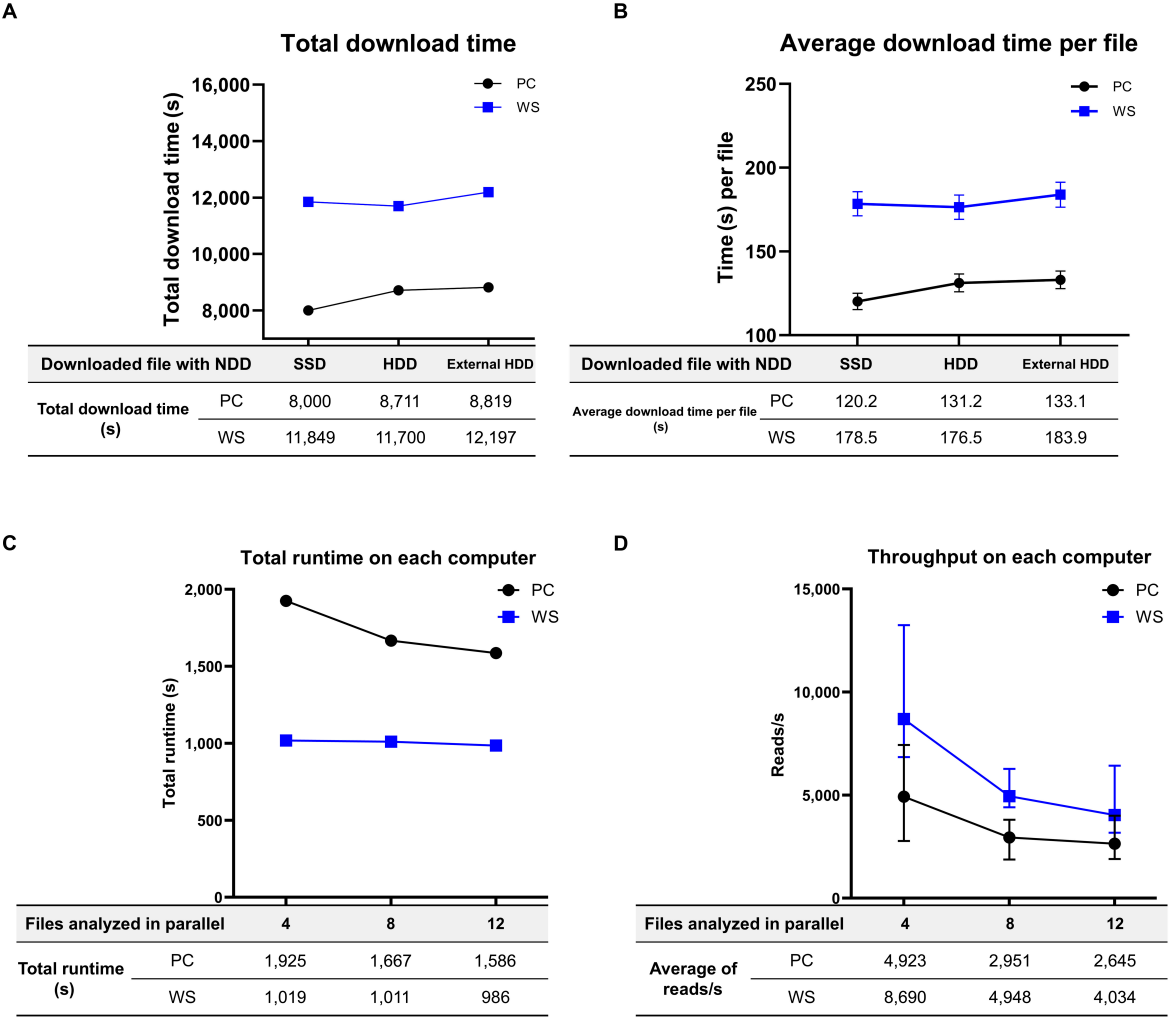
Comparison of NDD download performance and ISSM analysis performance on a personal computer (PC) and workstation (WS). Points indicate the mean and error bars show variability across files. The tables under each panel list the corresponding numeric values. (A) Total download time for 263 FASTQ files from PRJNA898830 using SSD, HDD, and an external HDD on the PC and the WS. (B) Average download time per file under the same conditions. For both systems, SSD was faster than HDD and the external HDD. (C) Total ISSM runtime when analyzing 4, 8, and 12 files in parallel on each system. (D) ISSM throughput measured as reads per second per file for the same parallel settings.

Using the SSD, HDD, and an external HDD on both the PC and WS, we downloaded the same 263 FASTQ files from PRJNA898830. Total download times on the PC were 2 h 13 min 20 s on SSD, 2 h 25 min 11 s on HDD, and 2 h 26 min 59 s on the external HDD. On the WS, totals were 3 h 17 min 29 s on SSD, 3 h 15 min 00 s on HDD, and 3 h 23 min 17 s on the external HDD. The mean total duration was therefore 2 h 21 min 50 s on the PC and 3 h 18 min 35 s on the WS (Fig. 3A).

Average download time per file showed the same trend. The PC was faster than the WS across all storage types, and for both systems the order was SSD faster than HDD faster than external HDD (Fig 3B). Compared to the WS, the PC completed the batch faster by 1 h 04 min 09 s on SSD, 49 min 49 s on HDD, and 56 min 18 s on the external HDD (Table S1).

Although the WS has more CPU cores and larger RAM, the PC provides higher single-core performance. The NDD processes one run per worker and executes up to 4 workers in parallel. Within each worker, fasterq-dump and subsequent gzip compression were run to completion for that file. In this configuration, time is driven by per-core speed and disk input/output, which likely explains why the PC completed the batch faster.

### Scalability and runtime evaluation of the ISSM using parallel file processing

The ISSM can be executed in parallel processing mode, which users can enable by selecting the “Use custom parallel processing” option in the ISSM menu. On a desktop PC, total analysis times were recorded as 32 min with 4 parallel files, 28 min with 8 files, and 26 min with 12 files. These results indicate that analysis speed improved as the number of simultaneously processed files increased.

In contrast, on the WS, the total analysis times were 17 min (4 files), 17 min (8 files), and 16 min (12 files). Unlike the PC, the WS showed no substantial variation in total analysis time; however, it consistently outperformed the PC due to its superior hardware specifications (Fig. 3C).

The number of reads processed per second per file was also measured. On the PC, processing speeds were 4,923 reads/s (4 files), 2,951 reads/s (8 files), and 2,645 reads/s (12 files). On the WS, the corresponding values were 8,690 reads/s, 4,948 reads/s, and 4,034 reads/s, respectively (Fig. 3D and Table S2).

### ISSM analysis of a FASTA file containing diverse coronavirus genomic sequences using published coronavirus-targeting primer- and probe-derived sequences

We analyzed a FASTA file containing approximately 34,899 coronavirus genomic sequences, including Alphacoronavirus 1 and SARS-CoV-2, using 114 probe sequences derived from published primers or probes together with 5 newly designed probe sequences in ISSM, as summarized in Table 2.

**Fig. 4.**
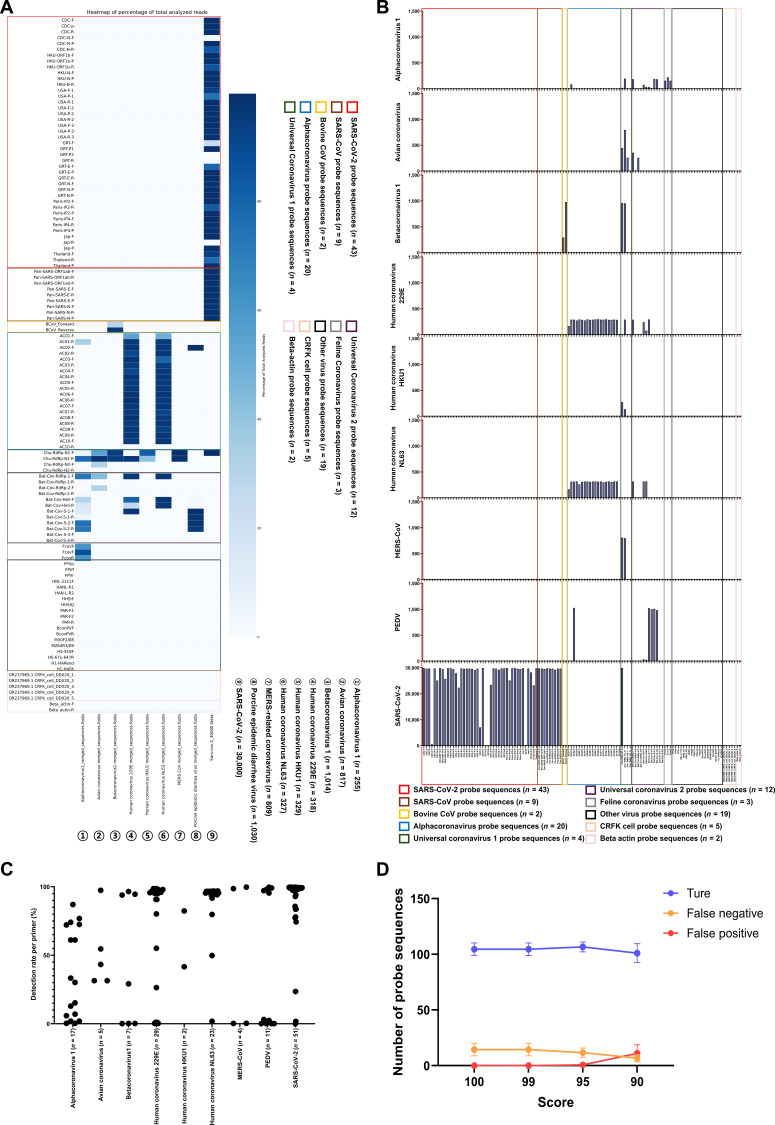
ISSM analysis of various coronavirus-group-specific FASTA datasets using multiple coronavirus-group-specific probe sequences, other viral detection probes, CRFK cell line sequences, and beta-actin probes. (A) Default heatmap output generated by the ISSM. Probe sequences are color coded based on their target group: SARS-CoV-2 (red), SARS-CoV (brown), Bovine CoV (yellow), Alphacoronavirus (blue), Universal coronavirus (green), Bat-CoV (purple), Feline coronavirus (gray), other viruses (black), CRFK cell (light orange), and beta-actin (pink). Color intensity represents the relative number of matched sequences, with deeper red indicating a higher number of matched sequences. (B) Bar graph showing the number of matched sequences per probe sequence for each coronavirus FASTA group. (C) Detection-rate graph of probes matched to each coronavirus FASTA group. The *x*-axis indicates the FASTA group and the number of probes with matches, and the *y*-axis represents the detection rate. (D) Graph showing the trend of detection specificity as the “Percentage of match to the probe sequence” parameter in ISSM is adjusted to 100, 99, 95, and 90, indicating how probe accuracy changes with different stringency thresholds. MERS-CoV, Middle East respiratory syndrome-related coronavirus; PEDV, porcine epidemic diarrhea virus.

When 93 coronavirus-targeting probe sequences were evaluated with the “Percentage of match to the probe sequence” option set to 100%, they successfully identified their intended targets, as illustrated in the heatmap visualization (Fig. 4A). No false positives were observed for any of the evaluated probe sets. The number of false negatives varied between 7 and 25, depending on the specific probe set used (Fig. 4B, Fig. S1A, and Table S3).

A closer examination of group-specific probe sequences revealed that each group was detected by at least one probe sequence derived from the same reference study. For the universal probe sequence sets, Set 1 enabled detection across all coronavirus groups through at least one probe sequence. However, Set 2 failed to detect sequences from the Human coronavirus HKU1 and SARS-CoV-2 groups. Detection rates of the genomic sequences identified by the probes ranged from 0.01% to 99.75%. Among the various groups, Avian coronavirus and Human coronavirus HKU1 exhibited the highest minimum detection rates, at 31.46% and 41.64%, respectively. In contrast, the minimum detection rates for the remaining groups ranged from 0.01% to 0.37%. Maximum detection rates ranged from 82.37% to 99.75%, indicating that the efficiency of target detection can vary substantially depending on the specific probe sequence employed, even within the same viral group (Fig. 4C and Table S4).

Given that the primers were originally designed for PCR applications, they were expected to tolerate some sequence mismatches while still enabling detection. To assess this, matching thresholds were systematically adjusted to 99%, 95%, and 90%. At a 99% threshold, results closely mirrored those obtained with a 100% match. Lowering the threshold to 95% led to increased target detection and a reduction in false negatives; however, some false positives began to appear. At a 90% threshold, the number of accurate detections (true positives, true negatives, and valid optional targets) declined significantly, while false positives increased sharply (Fig. 4D, Fig. S1A to C, and Table S3).

These findings suggest that, when employing the ISSM program with previously published PCR primers or probe sequences, a threshold of 95% or higher is recommended to achieve an optimal balance between sensitivity and specificity.

### Performance validation of the ISSM using coronavirus FASTQ datasets generated in CRFK cells

**Fig. 5.**
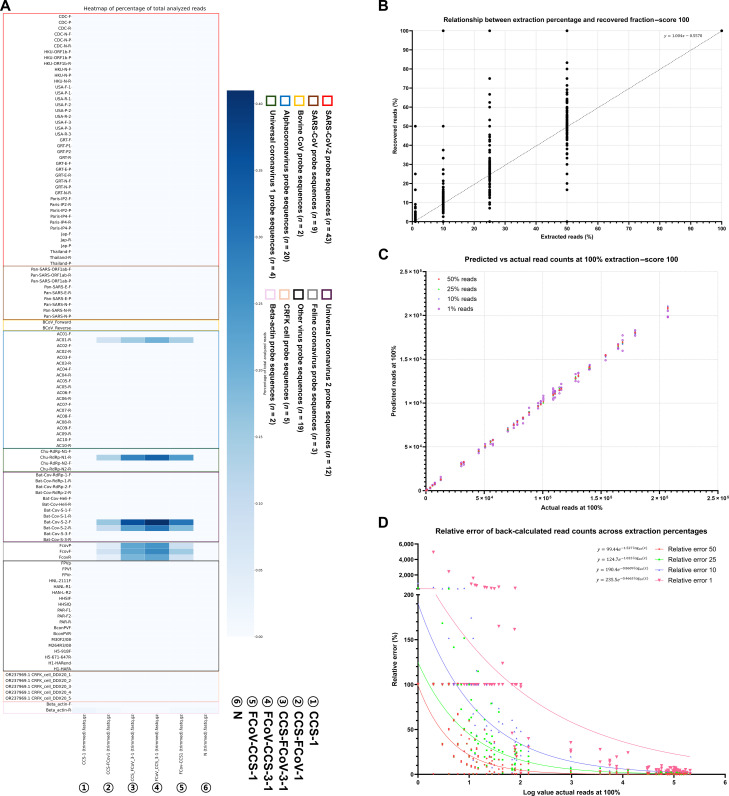
ISSM analysis of an NGS dataset obtained from CRFK cells infected with feline coronavirus (FCoV), using probes targeting various coronavirus groups, other viruses, CRFK cell sequences, and beta-actin. (A) Heatmap output generated by the ISSM. Probe sequences are color coded according to their target: SARS-CoV-2 (red), SARS-CoV (brown), Bovine CoV (yellow), Alphacoronavirus (blue), Universal coronavirus (green), Bat-CoV (purple), Feline coronavirus (gray), other viruses (black), CRFK cell (light orange), and beta-actin (pink). Color intensity indicates the relative number of matched reads, with deeper red representing higher read counts. (B) Relationship between extraction fraction and detected matched read proportion. Matched read counts obtained at 1%, 10%, 25%, and 50% extraction were expressed as percentages relative to the corresponding matched read counts obtained under 100% extraction, and a linear regression trendline was fitted to model this relationship. (C) Back-calculated 100% equivalent matched read counts derived from the regression equation shown in panel (B), compared with the actual matched read counts obtained under 100% extraction. The actual 100% extraction values used as reference are provided in Table S5. (D) Reliability assessment of downsampling-based back-calculation. The relationship between the log-transformed actual matched read counts obtained under 100% extraction and the relative error of the back-calculated 100% equivalent values is shown for each extraction setting. Trend equations were used to estimate approximate matched read ranges corresponding to selected relative error thresholds, which are summarized in Table 5.

When analyzing the raw NGS dataset using the ISSM with both the “Percentage of match to the probe sequence” and “Percentage of reads to extract from the total reads” options set to 100, the resulting heatmap showed detection signals only from the beta-actin-related probes in both cell culture supernatant and negative control samples. In contrast, samples infected with FCoV exhibited signals not only for FCoV-specific probes but also for 4 universal coronavirus probes and 3 additional probes that were not specifically designed for FCoV but nevertheless showed potential cross-detection capability (Fig. 5A).

Further inspection of the raw output identified matched reads for 10 additional probes that were less prominent in the heatmap but nevertheless showed detectable signals. When quantifying the number of matched reads per probe across NGS files, all 3 FCoV-specific probes successfully detected reads in the FCoV-infected samples. Depending on the individual FCoV-infected dataset, 3 or 4 of the 4 universal coronavirus probes showed positive detection, and 9 or 10 cross-reactive probes also yielded matched reads.

False negatives were observed for 1 to 7 probes, and false positives generated only 1 to 22 matched reads per probe, a negligible amount compared to true positive probes, which yielded 1 to 168,373 matched reads (Fig. S2 and Table S5).

To further quantify the reliability of downsampling-based estimation, the matched read counts obtained under the 1%, 10%, 25%, and 50% extraction settings were normalized to the corresponding values obtained under 100% extraction, and the relationship between extraction fraction and detected proportion was examined (Fig. 5B and Table S5). A linear relationship was observed, and the fitted regression equation was y=1.004x−0.5570.

Using this regression equation, matched read counts obtained under each extraction setting were converted into 100% equivalent estimates and compared with the actual matched read counts obtained under 100% extraction (Fig. 5C). The discrepancy between the back-calculated values and the actual 100% values was then expressed as relative error and analyzed against the log-transformed actual matched read counts at 100% extraction (Fig. 5D and Table 5). Across extraction fractions, larger full-data matched read counts were generally associated with lower relative error, indicating that the reliability of downsampling-based back-calculation improved as target abundance increased. The accompanying table provides approximate matched read ranges corresponding to selected relative error thresholds for each extraction setting.

**Table 5.** Approximate matched-read ranges corresponding to selected relative error thresholds for each extraction setting, based on the fitted relationships shown in Fig. 5D

Relative error threshold	Back-calculated 100% reads from 50% extraction	Back-calculated 100% reads from 25% extraction	Back-calculated 100% reads from 10% extraction	Back-calculated 100% reads from 1% extraction
	$y=99.44e^{-1.527\log10\left(x\right)}$	$y=124.7e^{-1.023\log10\left(x\right)}$	$y=190.4e^{-0.8609\log10\left(x\right)}$	$y=235.5e^{-0.4663\log10\left(x\right)}$
1%	1,028	52,169	1,250,994	516,000,000,000
5%	91	1,394	16,896	182,541,572
10%	32	293	2,646	5,954,955
20%	11	62	414	194,265

These results provide an empirical guide for interpreting downsampling-based estimation in the present FCoV-related dataset. However, these values should not be interpreted as universal cutoffs applicable to all datasets or probe sets. Rather, they indicate that the practical reliability of downsampling depends on both extraction fraction and full-data matched read abundance. Accordingly, 100% extraction remains the recommended default setting when target abundance is unknown.

### ISSM-based detection and subtyping of HIV-1 in clinical FASTQ datasets

**Fig. 6.**
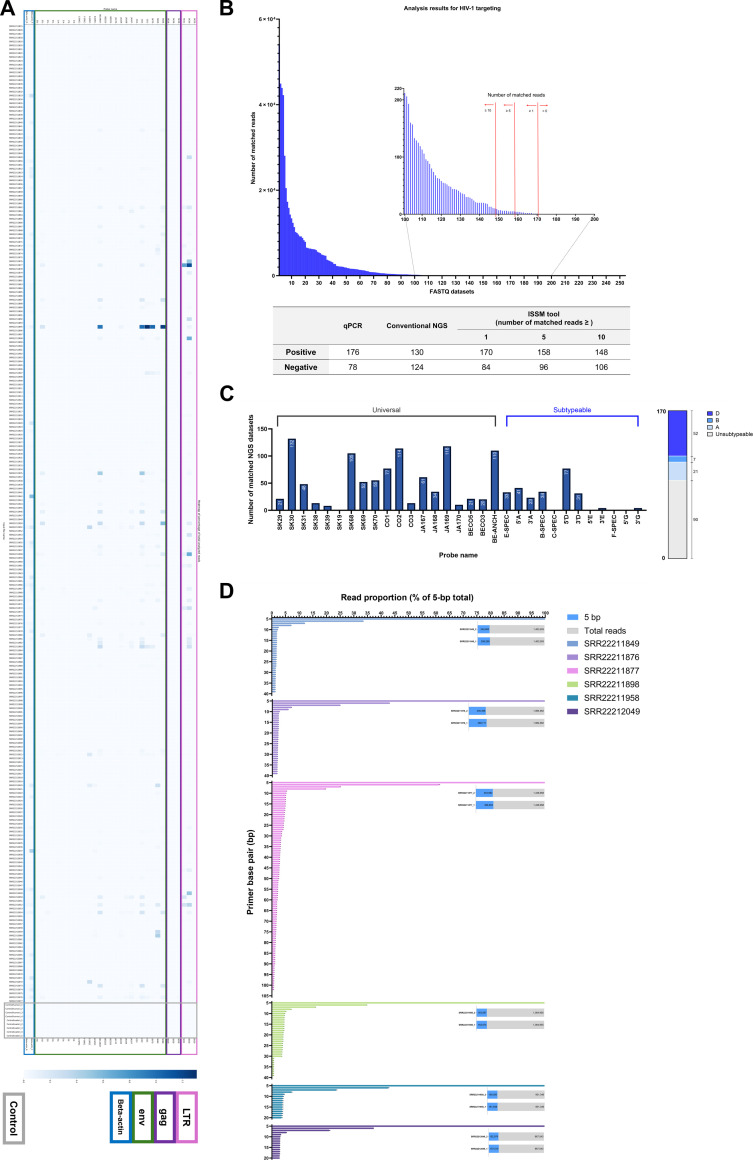
Analysis of 263 HIV-related NGS datasets using the ISSM. (A) Heatmap representing the analysis results of all 263 datasets, including 9 control samples. Probe sequences are color coded based on their target gene: LTR (pink), gag (purple), env (green), and beta-actin (blue). Color intensity indicates the relative number of matched reads, with darker shades representing higher read counts. (B) Bar graph summarizing ISSM detection of the 263 HIV-related datasets under 3 read-count criteria (≥1, ≥5, and ≥10 matched reads), excluding datasets matched only to the beta-actin probe. The thresholds of ≥5 and ≥10 were used as more conservative sensitivity-analysis cutoffs than a single-read criterion, rather than as universally validated diagnostic thresholds. This panel is presented to illustrate how screening interpretation changes as low-count matches are progressively excluded. (C) Graph showing the number of datasets with ≥1 matched read for each HIV-1 target probe. Probes capable of distinguishing HIV-1 subtypes A to D, F, G, and CRF01_AE are labeled as “subtypeable”. Based on matches to these subtypeable probes, subtype assignments for the 170 suspected HIV-1-positive datasets are summarized in the right-hand bar graph. (D) Graph showing the pattern of matched read counts for the extended SK30 probe across probe lengths from 5 to 105 bp in 6 paired datasets. Matched read counts at each probe length are presented as proportions relative to the 5-bp matched read count, which was set to 100%. The bar graph indicates the proportion of 5-bp matched reads among the total reads in each FASTQ file.

A total of 263 NGS datasets were analyzed using HIV-1-targeting probes. In the control datasets, no reads matched any HIV-1 probes, confirming specificity (Fig. 6A and Table S6). Among the universal HIV-1 probes, those targeting the LTR and env genes exhibited higher match rates compared to the probe targeting the gag gene.

Of the 263 datasets, 170 showed at least one matched read, suggesting probable HIV-1 infection under the most permissive screening criterion. To examine the effect of low-count matches on screening interpretation, ISSM results were additionally summarized using read-count thresholds of ≥1, ≥5, and ≥10 matched reads (Fig. 6B). The thresholds of ≥5 and ≥10 were used as more conservative sensitivity-analysis cutoffs than a single-read criterion, rather than as universally validated diagnostic thresholds. As the threshold increased, low-count matches were progressively excluded, yielding a more conservative interpretation while the main detection pattern remained broadly consistent. Because the ISSM and the Chan Zuckerberg ID v6.6 pipeline use different analytical objectives and positivity criteria, this comparison should be interpreted as a contextual reference rather than a direct sensitivity benchmark. Among these 170 suspected HIV-1-positive datasets, probe SK30 (a universal probe) was the most frequently detected, with matches in 132 datasets, while SK19 showed no matches (Fig. 6C).

Subtype analysis using subtype-specific probes revealed that only 80 datasets could be assigned a likely HIV-1 subtype. The remaining 90 either had no match with any subtype-specific probe or matched multiple subtypes, preventing definitive classification. Among the 80 subtypeable datasets, 3 subtypes, A, B, and D, were identified, with subtype D being the most prevalent (*n* = 52).

To further examine the effect of probe length on matched read counts, matched read counts at different probe lengths ranging from 5 to 105 bp were analyzed in 6 paired datasets with high matched read counts for SK30 (17 bp) (Fig. 6D). The highest matched read count was observed at the shortest probe length, 5 bp (Table S7), which was therefore set as the reference value (100%) to express the matched read counts at the other probe lengths as relative percentages. These relative values decreased as probe length increased and then plateaued within a certain length range. Notably, the plateau region included probe lengths commonly used for PCR probes (approximately 20 bp). These findings suggest that appropriate optimization of probe length may improve detection efficiency.

To evaluate whether suspected HIV-1-positive datasets could be detected using downsampled datasets, the 170 suspected HIV-1-positive datasets were re-analyzed using sampling rates of 1%, 3%, 5%, and 10%. Each condition was tested in triplicate, and the number of datasets with zero matched reads was recorded (Fig. S3A). As the sampling rate decreased, the number of datasets with no matches increased, and the frequency of datasets showing zero detection in all 3 replicates also rose, indicating reduced detection sensitivity at lower sampling levels (Fig. S3B).

These findings support the use of downsampling only as an optional resource-saving strategy, whereas 100% extraction remains preferable when the abundance of target-positive reads is unknown.

### Detection of KRAS mutations in colorectal cancer NGS data using the ISSM

**Fig. 7.**
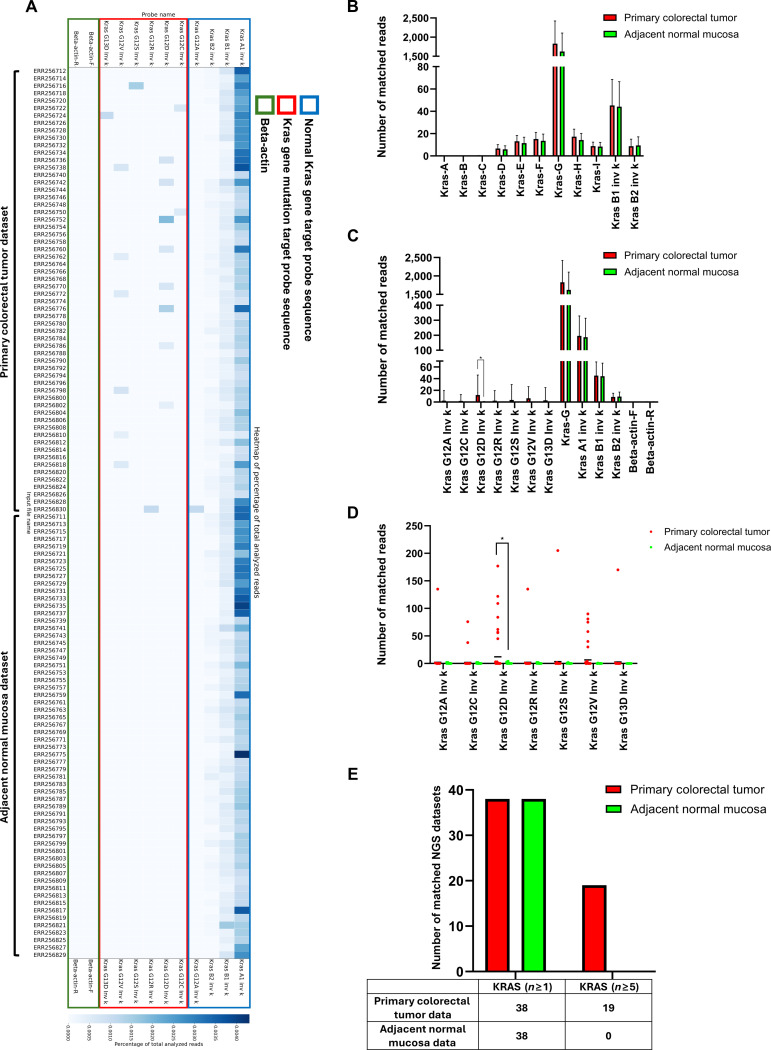
Analysis of 60 primary colorectal tumor NGS datasets and 60 adjacent normal mucosa NGS datasets using the ISSM with probe sequences targeting the KRAS gene and KRAS mutations. (A) Heatmap generated by the ISSM showing relative read counts matched to each probe; darker colors indicate higher numbers of matched reads. (B) Bar graph comparing the average number of matched reads for KRAS-A to KRAS-I probes (designed from the KRAS-wild-type sequence) and validated reference probes (KRAS B1 inv k and KRAS B2 inv k). Red bars represent tumor datasets, and green bars represent normal datasets. (C) Comparison of average matched read counts among KRAS-mutation-specific probes, the KRAS-G probe, and negative control probes. (D) Dot plot showing matched read counts for KRAS-mutation-specific probes only. (E) Comparison of the number of datasets where KRAS-mutation probes matched at least 1 read versus 5 or more reads.

Heatmap analysis using KRAS gene probes revealed that among the 60 primary colorectal tumor tissue NGS datasets, mutation-specific probes detected signals in 18 samples. Specifically, mutations were detected as follows: G12A (1 case), G12C (2 cases), G12D (8 cases), G12R (1 case), G12S (1 case), G12V (6 cases), and G13D (1 case). One primary colorectal tumor tissue sample exhibited signals for both G12A and G12R probes. In contrast, none of the 60 adjacent normal mucosal tissue datasets showed matches with any mutation probes (Fig. 7A and Table S8). However, because the reference study used tumor-adjacent normal mucosa rather than an independent healthy-control cohort, the paired colorectal dataset was used here as a comparative screening example rather than as a definitive mutation benchmark.

The detection performance of KRAS-wild-type-targeting probes (KRAS-A to KRAS-I) was compared to validated control probes (KRAS B1 inv k and B2 inv k). Among these, KRAS-G showed the highest average number of matched reads, exceeding that of the control probes (Fig. 7B and Table S9).

We further compared the mean number of matched reads between primary colorectal tumor tissue and adjacent normal mucosal tissue samples for each probe. No significant difference was observed for wild-type or negative control probes. However, mutation-specific probes yielded significantly higher read counts in primary colorectal tumor tissue samples. In particular, the KRAS G12D mutation probe showed a statistically significant difference, with a *P* value of 0.009341, based on a 2-tailed *t* test (Fig. 7C and D and Table S10).

When comparing the matched sample counts between the 60 primary colorectal tumor tissue and 60 adjacent normal mucosal tissue datasets, 38 primary colorectal tumor tissue samples (63.3%) and 38 adjacent normal mucosal tissue samples (63.3%) showed at least one matched read for any KRAS mutation probe. However, applying a stricter threshold of 5 or more matched reads, only 19 primary colorectal tumor tissue samples (31.7%) were considered positive, while none of the adjacent normal mucosal tissue samples met this criterion (Fig. 7E and Table S10). Additional threshold-dependent exploratory analysis showed that increasing the matched-read cutoff improved specificity and precision at the cost of reduced sensitivity, with a relatively balanced trade-off observed at thresholds of ≥3 to 4 matched reads (Table S11). Because the source cohort consisted of paired primary tumor and adjacent normal mucosa specimens rather than an independent KRAS-mutant versus KRAS-wild-type benchmark dataset, these metrics should be interpreted as exploratory surrogate estimates rather than definitive diagnostic performance measures.

## Discussion

NGS-based metagenomic analysis and diagnostics have garnered increasing attention for their potential in clinical applications. While NGS technologies continue to evolve in terms of scale and affordability, downstream analysis remains a significant bottleneck, particularly in clinical and diagnostic settings. This is especially true for viral detection, where the complexity of taxonomic assignments, the need for continual database updates, and the lack of standardized analysis pipelines have hindered the clinical adoption of NGS [55]. To address these challenges, we developed 2 tools, the ISSM and NDD, aimed at facilitating virus detection and targeted sequence mining through probe-based, sample-specific NGS screening with minimal technical barriers.

The NDD provides a graphical interface that simplifies the retrieval of public NGS datasets from repositories such as the NCBI SRA and the European Molecular Biology Laboratory–ENA. Users can easily search for, select, and download sequencing data in FASTQ or FASTA format—without the need for command-line operations or complex environment configurations. The batch-download feature, including associated metadata, is particularly useful for re-analysis or comparative studies using global NGS datasets.

The ISSM enables rapid and direct detection of specific nucleotide sequences in FASTA or NGS datasets using probe sequences, such as existing PCR primers (including degenerate ones), hybridization probes, or custom-designed probe sequences. Unlike traditional NGS analysis approaches [56,57], the ISSM does not rely on prebuilt sequence databases. Instead, it utilizes user-defined probe sequence lists in FASTA format—supporting more than 100 probes as demonstrated in this study. Since database-based approaches require constant updates and curation [58], the use of customizable probe lists provides an efficient alternative for users seeking specific target sequences within NGS datasets. The intuitive GUI further enhances accessibility and usability, particularly for users with limited coding experience or without access to high-performance computing environments. Beyond detection, the ISSM enables the retrieval of matched read sequences, allowing users to validate hits through external alignment tools or further characterize the genomic context. This feature is particularly valuable for clinical applications, where verification of mutation or pathogen identity is critical, and supports integration into broader diagnostic or research workflows.

For mutation-oriented applications such as KRAS screening, probe matches should be interpreted as preliminary candidate signals that require alignment-based follow-up analysis to confirm whether the detected reads map to the expected genomic locus. In addition, threshold-dependent performance summaries derived from paired tumor-adjacent normal datasets should be interpreted as exploratory surrogate evaluations since such cohorts do not constitute formal KRAS-mutant versus KRAS-wild-type diagnostic benchmark sets.

The conceptual contribution of the ISSM should be understood primarily as workflow accessibility rather than as a fundamentally new short-sequence search algorithm. Existing command-line tools based on k-mer matching, seed-based filtering, or direct read searching can also identify short-sequence motifs in sequencing reads. In contrast, the ISSM was designed as a GUI-based probe-screening workflow that allows non-bioinformaticians to apply short user-defined probes directly to raw FASTQ/FASTA datasets without database construction as an initial step. Fuzzy matching was selected to support flexible approximate matching of short probe sequences in a user-configurable manner, although positive signals should still be interpreted as preliminary screening results requiring confirmatory downstream analysis.

The ISSM incorporates a sampling-based strategy that balances speed and accuracy for large datasets, enabling repeated subsampling-based analyses when needed. In the FCoV-related dataset, additional error-based analysis across reduced extraction fractions showed that the reliability of downsampling-based estimation depends on both extraction fraction and full-data matched read abundance, providing an empirical basis for interpreting reduced-extraction results. Accordingly, downsampling should be regarded as a dataset-dependent auxiliary strategy for preliminary screening, whereas full (100%) extraction remains the preferred default when target abundance in raw datasets is unknown. This feature may still be advantageous in resource-limited or high-throughput settings where rapid preliminary triage is needed. Additionally, the ISSM was useful for identifying HIV-1 subtypes, for preliminary KRAS mutation-associated probe screening in colorectal cancer datasets, and for detecting various coronaviruses using more than 100 probe sequences, highlighting its broad applicability across diverse NGS-based clinical diagnostic fields. As shown in Figs. 4 and 5, individual probe sequences exhibited different detection efficiencies, likely owing to variation in target-site representation among sequenced reads. Therefore, combining multiple probe sequences for the same target may improve the robustness of preliminary screening interpretation. Because the ISSM and the Chan Zuckerberg ID differ in analytical objective and positivity criteria, the comparison between the 2 approaches should be interpreted as a contextual reference rather than a direct sensitivity benchmark.

In conclusion, the ISSM and NDD provide accessible, practical solutions for clinicians and researchers seeking to leverage NGS datasets for targeted sequence detection. By enabling efficient use of public NGS resources and existing primer/probe sets, the ISSM supports clinical diagnostics in a wide range of applications, including infectious disease and oncology. Its unique combination of probe-based fuzzy matching and user-friendly design bridges the gap between complex bioinformatics tools and routine diagnostic needs, thereby advancing the integration of NGS technologies into real-world clinical practice.

We used a Windows-only packaged executable in this study, and no Linux or macOS build is available in the current release. According to StatCounter’s worldwide desktop operating system (OS) data covering August 2024 through August 2025, Windows held 69.75% during this period, which is consistent with our field experience where Windows is typically used in practice [59]. Although the screening logic itself is operating-system agnostic, providing Linux and macOS packages would improve access in mixed-OS laboratory environments, and we recognize this as a practical area for future work.

## Conclusion

In this work, we developed the NDD and ISSM, 2 complementary Windows-based applications that together provide an accessible workflow for public dataset retrieval and probe-based pre-alignment screening. The NDD reduces the technical barrier to accessing large-scale NGS data by providing a graphical interface for searching and selecting projects or runs from the NCBI SRA and ENA, and by automating metadata parsing, batch-download management, and real-time file conversion into analysis-ready formats. The ISSM complements the NDD with a scalable, probe-based sequence-mining engine that operates directly on FASTQ and FASTA files, allowing users to evaluate short nucleotide probes, including degenerate primer-/probe-derived sequences, without reference genome alignment or database preformatting.

Using representative virology and oncology datasets, including tens of thousands of coronavirus genomes, HIV-positive plasma samples, and colorectal tumor tissues, we showed that the combined NDD–ISSM workflow can achieve high specificity, stable runtime performance, and subtype-oriented screening and mutation-associated read discrimination on standard desktop hardware. These results demonstrate that investigators with limited bioinformatics expertise can perform targeted sequence mining, candidate primer and probe evaluation, and rapid hypothesis testing using existing NGS data, with minimal command-line interaction or custom scripting.

By lowering the practical and computational barriers to NGS data retrieval and in silico assay evaluation, the NDD and ISSM help bridge the gap between raw sequencing archives and routine diagnostic assay development. The framework presented here can be readily extended to additional pathogens, cancer panels, and other application areas where rapid, probe-based sequence screening is required. The software and source code are freely available for noncommercial use at https://github.com/khk1329/NDD-and-ISSM.git, enabling further validation, adaptation, and community-driven improvement.

## Ethical Approval

This study uses publicly available data originally published [52], under BioProject accession number PRJNA898830. According to the original publication, written informed consent was obtained from participants or their surrogates, and ethical approval was granted by the University of Virginia Institutional Review Board. This study includes a secondary analysis of publicly available data and did not involve new data collection from human subjects.

## Data Availability

The full source code, together with a standalone executable (.exe) version of the software, is publicly available at https://github.com/khk1329/NDD-and-ISSM.git. The executable file enables users to run the software without setting up the Python environment. The NGS datasets analyzed in this study are publicly available from the following repositories: NCBI Sequence Read Archive (SRA): feline coronavirus (FCoV)-infected CRFK cells: BioProject accession numbers [PRJNA1089373] (https://www.ncbi.nlm.nih.gov/bioproject/PRJNA1089373) and [PRJNA1090152] (https://www.ncbi.nlm.nih.gov/bioproject/PRJNA1090152); human immunodeficiency virus (HIV): BioProject accession number [PRJNA898830] (https://www.ncbi.nlm.nih.gov/bioproject/PRJNA898830). European Nucleotide Archive (ENA): tumor tissue samples: study accession [PRJEB1752] (https://www.ebi.ac.uk/ena/browser/view/PRJEB1752) (secondary accession: [ERP002442] (https://www.ebi.ac.uk/ena/browser/view/ERP002442)). All datasets were retrieved from public databases and processed using our custom software for downstream analysis.

## References

[1] Drossman H, Luckey JA, Kostichka AJ, D’Cunha J, Smith LM. High-speed separations of DNA sequencing reactions by capillary electrophoresis. Anal Chem. 1990;62(9):900–903. 2363514 10.1021/ac00208a003

[2] Luckey JA, Drossman H, Kostichka AJ, Mead DA, D’Cunha J, Norris TB, Smith LM. High speed DNA sequencing by capillary electrophoresis. Nucleic Acids Res. 1990;18(15):4417–4421. 2388826 10.1093/nar/18.15.4417 PMC331259

[3] Smith LM. High-speed DNA sequencing by capillary gel electrophoresis. Nature. 1991;349(6312):812–813. 2000151 10.1038/349812a0

[4] Margulies M, Egholm M, Altman WE, Attiya S, Bader JS, Bemben LA, Berka J, Braverman MS, Chen Y-J, Chen Z, et al. Genome sequencing in microfabricated high-density picolitre reactors. Nature. 2005;437(7057):376–380. 16056220 10.1038/nature03959 PMC1464427

[5] Shendure J, Porreca GJ, Reppas NB, Lin X, McCutcheon JP, Rosenbaum AM, Wang MD, Zhang K, Mitra RD, Church GM. Accurate multiplex polony sequencing of an evolved bacterial genome. Science. 2005;309(5741):1728–1732. 16081699 10.1126/science.1117389

[6] Bentley DR, Balasubramanian S, Swerdlow HP, Smith GP, Milton J, Brown CG, Hall KP, Evers DJ, Barnes CL, Bignell HR, et al. Accurate whole human genome sequencing using reversible terminator chemistry. Nature. 2008;456(7218):53–59. 18987734 10.1038/nature07517 PMC2581791

[7] Smith DR, Quinlan AR, Peckham HE, Makowsky K, Tao W, Woolf B, Shen L, Donahue WF, Tusneem N, Stromberg MP, et al. Rapid whole-genome mutational profiling using next-generation sequencing technologies. Genome Res. 2008;18(10):1638–1642. 18775913 10.1101/gr.077776.108 PMC2556265

[8] Wang Y, Zhao Y, Bollas A, Wang Y, Au KF. Nanopore sequencing technology, bioinformatics and applications. Nat Biotechnol. 2021;39(11):1348–1365. 34750572 10.1038/s41587-021-01108-x PMC8988251

[9] Kuczynski J, Lauber CL, Walters WA, Parfrey LW, Clemente JC, Gevers D, Knight R. Experimental and analytical tools for studying the human microbiome. Nat Rev Genet. 2011;13(1):47–58. 22179717 10.1038/nrg3129 PMC5119550

[10] Shapiro E, Biezuner T, Linnarsson S. Single-cell sequencing-based technologies will revolutionize whole-organism science. Nat Rev Genet. 2013;14(9):618–630. 23897237 10.1038/nrg3542

[11] Sorek R, Cossart P. Prokaryotic transcriptomics: A new view on regulation, physiology and pathogenicity. Nat Rev Genet. 2010;11(1):9–16. 19935729 10.1038/nrg2695

[12] Wang Z, Gerstein M, Snyder M. RNA-Seq: A revolutionary tool for transcriptomics. Nat Rev Genet. 2009;10(1):57–63. 19015660 10.1038/nrg2484 PMC2949280

[13] Gardy JL, Loman NJ. Towards a genomics-informed, real-time, global pathogen surveillance system. Nat Rev Genet. 2018;19(1):9–20. 29129921 10.1038/nrg.2017.88 PMC7097748

[14] Voelkerding KV, Dames SA, Durtschi JD. Next-generation sequencing: From basic research to diagnostics. Clin Chem. 2009;55(4):641–658. 19246620 10.1373/clinchem.2008.112789

[15] Gnirke A, Melnikov A, Maguire J, Rogov P, Le Proust EM, Brockman W, Fennell T, Giannoukos G, Fisher S, Russ C, et al. Solution hybrid selection with ultra-long oligonucleotides for massively parallel targeted sequencing. Nat Biotechnol. 2009;27(2):182–189. 19182786 10.1038/nbt.1523 PMC2663421

[16] Steiert TA, Fuß J, Juzenas S, Wittig M, Hoeppner MP, Vollstedt M, Varkalaite G, Abd HE, Brockmann C, Görg S, et al. High-throughput method for the hybridisation-based targeted enrichment of long genomic fragments for PacBio third-generation sequencing. NAR Genom Bioinform. 2022;4(3):lqac051. 35855323 10.1093/nargab/lqac051 PMC9278042

[17] Schwab TC, Perrig L, Göller PC, De Hoz FFG, Lahousse AP, Minder B, Günther G, Efthimiou O, Omar SV, Egger M, et al. Targeted next-generation sequencing to diagnose drug-resistant tuberculosis: A systematic review and meta-analysis. Lancet Infect Dis. 2024;24(10):1162–1176. 38795712 10.1016/S1473-3099(24)00263-9 PMC11881551

[18] Wylie TN, Wylie KM, Herter BN, Storch GA. Enhanced virome sequencing using targeted sequence capture. Genome Res. 2015;25(12):1910–1920. 26395152 10.1101/gr.191049.115 PMC4665012

[19] Zhan S, Jin H, Ji H, Hou X, Li J, Zhang Y, Zheng J, Cui L. Clinical diagnosis of Q fever by targeted next-generation sequencing for identification of Coxiella burnetii. BMC Infect Dis. 2025;25(1):190. 39920575 10.1186/s12879-024-10437-6 PMC11806902

[20] Wan JCM, Massie C, Garcia-Corbacho J, Mouliere F, Brenton JD, Caldas C, Pacey S, Baird R, Rosenfeld N. Liquid biopsies come of age: Towards implementation of circulating tumour DNA. Nat Rev Cancer. 2017;17(4):223–238. 28233803 10.1038/nrc.2017.7

[21] Menzel P, Ng KL, Krogh A. Fast and sensitive taxonomic classification for metagenomics with Kaiju. Nat Commun. 2016;7:11257. 27071849 10.1038/ncomms11257 PMC4833860

[22] Wood DE, Lu J, Langmead B. Improved metagenomic analysis with Kraken 2. Genome Biol. 2019;20(1):257. 31779668 10.1186/s13059-019-1891-0 PMC6883579

[23] Wood DE, Salzberg SL. Kraken: Ultrafast metagenomic sequence classification using exact alignments. Genome Biol. 2014;15(3):R46. 24580807 10.1186/gb-2014-15-3-r46 PMC4053813

[24] Liu H, Yin H, Li G, Li J, Wang X. Aperture: Alignment-free detection of structural variations and viral integrations in circulating tumor DNA. Brief Bioinform. 2021;22(6). 10.1093/bib/bbab290 34368852

[25] Newman AM, Bratman SV, Stehr H, Lee LJ, Liu CL, Diehn M, Alizadeh AA. FACTERA: A practical method for the discovery of genomic rearrangements at breakpoint resolution. Bioinformatics. 2014;30(23):3390–3393. 25143292 10.1093/bioinformatics/btu549 PMC4296148

[26] Webster J, Dang HX, Chauhan PS, Feng W, Shiang A, Harris PK, Pachynski RK, Chaudhuri AA, Maher CA. PACT: A pipeline for analysis of circulating tumor DNA. Bioinformatics. 2023;39(8). 10.1093/bioinformatics/btad489 PMC10415172 37549060

[27] Camacho C, Coulouris G, Avagyan V, Ma N, Papadopoulos J, Bealer K, Madden TL. BLAST+: Architecture and applications. BMC Bioinformatics. 2009;10:421. 20003500 10.1186/1471-2105-10-421 PMC2803857

[28] Narayanan S, Espindola AS, Malayer J, Cardwell K, Ramachandran A. Development and evaluation of microbe finder (MiFi)^®^: A novel in silico diagnostic platform for pathogen detection from metagenomic data. J Med Microbiol. 2023;72(6): 10.1099/jmm.0.001720. 10.1099/jmm.0.001720 37345698

[29] Bachmann M. RapidFuzz: A fast fuzzy string matching library for Python. GitHub. 13 February 2021. [accessed 7 May 2026] https://github.com/maxbachmann/RapidFuzz

[30] Yagyu F, Okitsu S, Tanamoto K, Ushijima H. Determination of HIV-1 subtypes (A-D, F, G, CRF01_AE) by PCR in the transmembrane region (gp41) with novel primers. J Med Virol. 2005;76(1):16–23. 15778948 10.1002/jmv.20318

[31] Ou CY, Kwok S, Mitchell SW, Mack DH, Sninsky JJ, Krebs JW, Feorino P, Warfield D, Schochetman G. DNA amplification for direct detection of HIV-1 in DNA of peripheral blood mononuclear cells. Science. 1988;239(4837):295–297. 3336784 10.1126/science.3336784

[32] Nijhuis M, Boucher CA, Schipper P, Leitner T, Schuurman R, Albert J. Stochastic processes strongly influence HIV-1 evolution during suboptimal protease-inhibitor therapy. Proc Natl Acad Sci USA. 1998;95(24):14441–14446. 9826719 10.1073/pnas.95.24.14441 PMC24392

[33] Yagyu F, Ikeda Y, Ariyoshi K, Sugiura W, Wongkhomthong SA, Masuda M, Ushijima H. Differentiation of subtypes B and E of human immunodeficiency virus type 1 by polymerase chain reaction using novel env gene primers. J Virol Methods. 2002;101(1-2):11–20. 11849679 10.1016/s0166-0934(01)00415-3

[34] Yu B, Xu C, Huang S, Ni J, Zhou J, Zhang Y, Wu M, Zhang J, Fang L. Development of a universal real-time RT-PCR assay for detection of pan-SARS-coronaviruses with an RNA-based internal control. Front Microbiol. 2023;14:1181097. 37275136 10.3389/fmicb.2023.1181097 PMC10232947

[35] Chu DKW, Pan Y, Cheng SMS, Hui KPY, Krishnan P, Liu Y, Ng DYM, Wan CKC, Yang P, Wang Q, et al. Molecular diagnosis of a novel coronavirus (2019-nCoV) causing an outbreak of pneumonia. Clin Chem. 2020;66(4):549–555. 32031583 10.1093/clinchem/hvaa029 PMC7108203

[36] U.S. Centers for Disease Control and Prevention. Research use only 2019-novel coronavirus (2019-nCoV) real-time RT-PCR primers and probes. CDC Stacks. 29 May 2020. [accessed 7 May 2026] https://stacks.cdc.gov/view/cdc/88834

[37] Corman VM, Landt O, Kaiser M, Molenkamp R, Meijer A, Chu DK, Bleicker T, Brünink S, Schneider J, Schmidt ML, et al. Detection of 2019 novel coronavirus (2019-nCoV) by real-time RT-PCR. Euro Surveill. 2020;25(3):2000045. 31992387 10.2807/1560-7917.ES.2020.25.3.2000045 PMC6988269

[38] World Health Organization. *Protocol: Real-time RT-PCR assays for the detection of SARS-CoV-2*. Paris: Institut Pasteur; 2020.

[39] Nao N, Shirato K, Katano H, Matsuyama S, Takeda M. Detection of second case of 2019-nCoV infection in Japan. Problem Set. 2020;2(23):1–9.

[40] Li D, Zhang J, Li J. Primer design for quantitative real-time PCR for the emerging coronavirus SARS-CoV-2. Theranostics. 2020;10(16):7150–7162. 32641984 10.7150/thno.47649 PMC7330846

[41] Cho YI, Kim WI, Liu S, Kinyon JM, Yoon KJ. Development of a panel of multiplex real-time polymerase chain reaction assays for simultaneous detection of major agents causing calf diarrhea in feces. J Vet Diagn Invest. 2010;22(4):509–517. 20622219 10.1177/104063871002200403

[42] Choi S, Kim KW, Ku KB, Kim S-J, Park C, Park D, Kim S, Yi H. Human Alphacoronavirus universal primers for genome amplification and sequencing. Front Microbiol. 2022;13: Article 789665. 35401489 10.3389/fmicb.2022.789665 PMC8990890

[43] Chamings A, Nelson TM, Vibin J, Wille M, Klaassen M, Alexandersen S. Detection and characterisation of coronaviruses in migratory and non-migratory Australian wild birds. Sci Rep. 2018;8(1):5980. 29654248 10.1038/s41598-018-24407-x PMC5899083

[44] Poon LLM, Chu DKW, Chan KH, Wong OK, Ellis TM, Leung YHC, Lau SKP, Woo PCY, Suen KY, Yuen KY, et al. Identification of a novel coronavirus in bats. J Virol. 2005;79(4):2001–2009. 15681402 10.1128/JVI.79.4.2001-2009.2005 PMC546586

[45] Gut M, Leutenegger CM, Huder JB, Pedersen NC, Lutz H. One-tube fluorogenic reverse transcription-polymerase chain reaction for the quantitation of feline coronaviruses. J Virol Methods. 1999;77(1):37–46. 10029323 10.1016/S0166-0934(98)00129-3 PMC7185542

[46] Cao N, Tang Z, Zhang X, Li W, Li B, Tian Y, Xu D. Development and application of a triplex TaqMan quantitative real-time PCR assay for simultaneous detection of feline calicivirus, feline parvovirus, and feline herpesvirus 1. Front Vet Sci. 2022;8: Article 792322. 35211534 10.3389/fvets.2021.792322 PMC8861203

[47] Arai S, Nguyen ST, Boldgiv B, Fukui D, Araki K, Dang CN, Ohdachi SD, Nguyen NX, Pham TD, Boldbaatar B, et al. Novel bat-borne hantavirus, Vietnam. Emerg Infect Dis. 2013;19(7):1159–1161. 23763849 10.3201/eid1907.121549 PMC3713973

[48] Sui X, Zhang X, Fei D, Zhang Z, Ma M. Simultaneous rapid detection of Hantaan virus and Seoul virus using RT-LAMP in rats. PeerJ. 2019;6: Article e6068. 30643674 10.7717/peerj.6068 PMC6329334

[49] Tong S, Chern SW, Li Y, Pallansch MA, Anderson LJ. Sensitive and broadly reactive reverse transcription-PCR assays to detect novel paramyxoviruses. J Clin Microbiol. 2008;46(8):2652–2658. 18579717 10.1128/JCM.00192-08 PMC2519498

[50] Pérez-Tris J, Williams RAJ, Abel-Fernández E, Barreiro J, Conesa JJ, Figuerola J, Martinez-Martínez M, Ramírez A, Benitez L. A multiplex PCR for detection of poxvirus and papillomavirus in cutaneous warts from live birds and museum skins. Avian Dis. 2011;55(4):545–553. 22312972 10.1637/9685-021411-Reg.1

[51] World Health Organization. *Protocols for influenza virus detection, February 2021*. Geneva (Switzerland): World Health Organization; 2021.

[52] Grundy BS, Parikh H, Jacob S, Banura P, Moore CC, Liu J, Houpt ER. Pathogen detection using metagenomic next-generation sequencing of plasma samples from patients with sepsis in Uganda. Microbiol Spectrum. 2023;11(1): Article e0431222. 10.1128/spectrum.04312-22 PMC9927450 36625651

[53] Thierry AR, Mouliere F, Messaoudi SE, Mollevi C, Lopez-Crapez E, Rolet F, Gillet B, Gongora C, Dechelotte P, Robert B, et al. Clinical validation of the detection of KRAS and BRAF mutations from circulating tumor DNA. Nat Med. 2014;20(4):430–435. 24658074 10.1038/nm.3511

[54] Han S-W, Kim H-P, Shin J-Y, Jeong E-G, Lee W-C, Lee K-H, Won J-K, Kim T-Y, Oh D-Y, Im S-A, et al. Targeted sequencing of cancer-related genes in colorectal cancer using next-generation sequencing. PLOS ONE. 2013;8(5): Article e64271. 23700467 10.1371/journal.pone.0064271 PMC3660257

[55] Chang W-S, Harvey E, Mahar JE, Firth C, Shi M, Simon-Loriere E, Geoghegan JL, Wille M. Improving the reporting of metagenomic virome-scale data. Commun Biol. 2024;7(1):1687. 39706917 10.1038/s42003-024-07212-3 PMC11662069

[56] Gu W, Deng X, Lee M, Sucu YD, Arevalo S, Stryke D, Federman S, Gopez A, Reyes K, Zorn K, et al. Rapid pathogen detection by metagenomic next-generation sequencing of infected body fluids. Nat Med. 2021;27(1):115–124. 33169017 10.1038/s41591-020-1105-z PMC9020267

[57] Simner PJ, Miller S, Carroll KC. Understanding the promises and hurdles of metagenomic next-generation sequencing as a diagnostic tool for infectious diseases. Clin Infect Dis. 2018;66(5):778–788. 29040428 10.1093/cid/cix881 PMC7108102

[58] Chorlton SD. Ten common issues with reference sequence databases and how to mitigate them. Front Bioinform. 2024;4:1278228. 38560517 10.3389/fbinf.2024.1278228 PMC10978663

[59] StatCounter Global Stats. Browser market share worldwide, August 2024–August 2025. StatCounter Global Stats. 1 September 2025. [accessed 7 May 2026] https://gs.statcounter.com/os-market-share/desktop/worldwide

