# Supplementary material for: Next-Generation Sequencing Dataset Downloader and In Silico Sequence Mining: Graphical-User-Interface-Based Tools for Accessible, Multiprobe Target Mining in Next-Generation Sequencing Data
Source: Comput Struct Biotechnol J. 2026 May 14;35(1):0095. doi: 10.34133/csbj.0095 (PMC13172811; doi:10.34133/csbj.0095)
Supplement: Supplementary 1 — Figs. S1 to S3 Tables S1 to S11 [file csbj.0095.f1.zip › sm.docx]

**Supplementary Data**





**Supplementary Fig S1.** Figure of the performance of probes targeting each coronavirus type. Blue indicates true positive and true negative detections, whereas orange and red represent false negatives and false positives, respectively. (A) Results obtained with the ISSM “Percentage of match to the probe sequence” parameter set to 100%. (B) Results obtained with the parameter set to 95%. (C) Results obtained with the parameter set to 90%.





**Supplementary Fig S2.** Sample-wise bar graphs showing matched read counts for probe sequences across the six CRFK-cell-related NGS datasets used in the FCoV validation experiment, including infected samples, CCS samples, and the negative control.


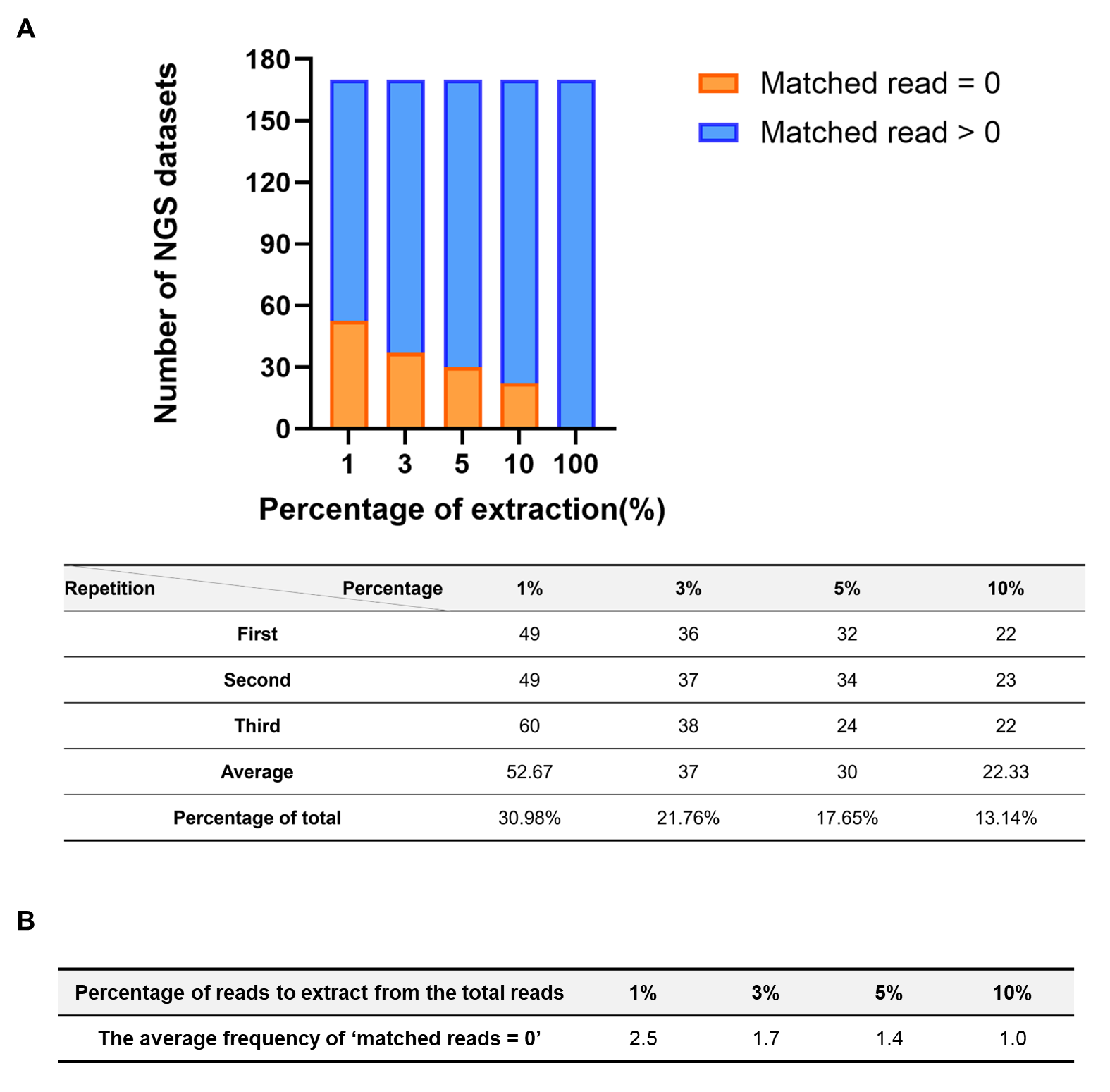


**Supplementary Fig S3.** Comparison of the number of datasets with zero matched reads according to the “Percentage of reads to extract from the total reads” setting among 170 suspected HIV-1 positive datasets. The 170 suspected HIV-1 positive datasets were those with non-zero matched reads among the 263 datasets in BioProject PRJNA898830 when analyzed using ISSM with 33 published HIV-1 probes, with both the “Percentage of reads to extract from the total reads” and “Percentage of match to the probe sequence” set to 100. (A) Graph showing the number of datasets with zero matched reads at “Percentage of reads to extract from the total reads” settings of 1, 3, 5, and 10. Results from three replicates for each percentage are summarized in the table. (B) Table summarizing, for each individual NGS dataset, the number of replicates (out of three) in which matched reads were zero at each extraction percentage.

**Supplementary Table S1.** Per-run download metrics for PRJNA898830 using NDD on a PC and a WS. For each of the 263 runs, the table lists run accession, file capacity in MB, total reads, and download time in seconds on the PC and on the WS across three storage paths SSD, HDD, and external HDD. The bottom rows summarize the minimum and maximum per-file times, the average per-file time, and the total download time including the h:m:s conversion for each storage path.

**Supplementary Table S2.** ISSM analysis results on a personal computer and workstation with 4, 8, and 12 files analyzed in parallel. The table includes the number of reads and file sizes for each dataset, along with the analysis time and the number of reads processed per second under each condition.

**Supplementary Table S3.** Table showing the number of probe sequences that successfully detected each coronavirus type using ISSM. Results are presented for four different thresholds of the “Percentage of match to the probe sequence” parameter: 100%, 99%, 95%, and 90%. Blue-highlighted cells indicate true positives, and orange-highlighted cells indicate false positives. False negatives were calculated as the total number of probes assigned to each target minus the number of true positives.

**Supplementary Table S4.** Table showing the number of sequences detected by each probe sequence for different coronavirus groups using ISSM with the “Percentage of match to the probe sequence” option set to 100, 99, 95, and 90.

**Supplementary Table S5.** Matched read counts for probe sequences across six CRFK-cell-related NGS datasets used in the FCoV validation experiment, including infected samples, CCS samples, and the negative control, analyzed under 100%, 50%, 25%, 10%, and 1% extraction settings. For sampled settings below 100%, three independent runs are shown.

**Supplementary Table S6.** Table showing the number of matched reads for 33 published HIV-1 probe sequences across 263 HIV-related NGS datasets from BioProject PRJNA898830, analyzed using ISSM with both the “Percentage of reads to extract from the total reads” and “Percentage of match to the probe sequence” set to 100. For each dataset, matched read counts were calculated as the sum of matched reads from each paired FASTQ file. Probe sequences are grouped according to their target region as LTR (pink), gag (purple), env (green), and beta-actin (blue).

**Supplementary Table S7.** Table showing the number of reads matched to the 5-bp region of the HIV-1 probe sequence SK30 across the six datasets with the highest matched read counts for SK30 among 263 HIV-related datasets from BioProject PRJNA898830, analyzed using ISSM with both the “Percentage of reads to extract from the total reads” and “Percentage of match to the probe sequence” set to 100.

**Supplementary Table S8.** Table showing the number of matched reads across NGS datasets from BioProject PRJEB1752, including 60 primary tumor samples and 60 adjacent normal samples, analyzed using ISSM with two beta-actin probes (Beta-actin-F and Beta-actin-R), three published reference probes (KRAS A1, KRAS B1, and KRAS B2), and seven KRAS mutation targeting probes (G12A, G12C, G12D, G12R, G12S, G12V, and G13D). Both the “Percentage of reads to extract from the total reads” and “Percentage of match to the probe sequence” were set to 100. For each dataset, matched read counts were calculated as the sum of matched reads from each paired FASTQ file.

**Supplementary Table S9.** Table showing the number of matched reads across NGS datasets from BioProject PRJEB1752, including 60 primary tumor samples and 60 adjacent normal samples, analyzed using ISSM with nine user designed KRAS wild type probes (KRAS-A to KRAS-I) and two published reference probes (KRAS B1 and KRAS B2). Both the “Percentage of reads to extract from the total reads” and “Percentage of match to the probe sequence” were set to 100. For each dataset, matched read counts were calculated as the sum of matched reads from each paired FASTQ file.

**Supplementary Table S10.** Table showing the number of matched reads across NGS datasets from BioProject PRJEB1752, including 60 primary tumor samples and 60 adjacent normal samples, analyzed using ISSM with seven user designed KRAS mutation targeting probes (KRAS G12A, G12C, G12D, G12R, G12S, G12V, and G13D), one user designed KRAS wild type probe (KRAS-G), three published reference probes (KRAS A1, KRAS B1, and KRAS B2), and two beta-actin probes (Beta-actin-F and Beta-actin-R). Both the “Percentage of reads to extract from the total reads” and “Percentage of match to the probe sequence” were set to 100. For each dataset, matched read counts were calculated as the sum of matched reads from each paired FASTQ file. For each probe, p-values were obtained from t-tests comparing matched read counts between paired tumor and normal tissue datasets from the same patient.

**Supplementary Table S11.** Threshold dependent exploratory performance summary for KRAS mutation oriented screening in the colorectal tumor adjacent normal paired dataset from Han et al. Because the source cohort consisted of paired primary tumor and adjacent normal mucosa specimens, and KRAS alterations were reported in only a subset of tumors in the original study, these values should be interpreted as surrogate exploratory estimates rather than definitive diagnostic performance measures.
